# The histone demethylase PHF8 regulates TGFβ signaling and promotes melanoma metastasis

**DOI:** 10.1126/sciadv.abi7127

**Published:** 2022-02-18

**Authors:** Rana S. Moubarak, Ana de Pablos-Aragoneses, Vanessa Ortiz-Barahona, Yixiao Gong, Michael Gowen, Igor Dolgalev, Sorin A. A. Shadaloey, Diana Argibay, Alcida Karz, Richard Von Itter, Eleazar Carmelo Vega-Sáenz de Miera, Elena Sokolova, Farbod Darvishian, Aristotelis Tsirigos, Iman Osman, Eva Hernando

**Affiliations:** 1 Department of Pathology, NYU School of Medicine, New York, NY 10016, USA.; 2 Interdisciplinary Melanoma Cooperative Group, NYU Cancer Institute, New York, NY 10016, USA.; 3 Laura and Isaac Perlmutter Cancer Center, NYU Langone Health, New York, NY 10016, USA.; 4 Applied Bioinformatics Laboratories, NYU School of Medicine, NY 10016, USA.; 5 NYU School of Medicine Institute for Computational Medicine, New York, NY 10016, USA.; 6 Ronald O. Perelman Department of Dermatology, NYU School of Medicine, New York, NY 10016, USA.

## Abstract

The contribution of epigenetic dysregulation to metastasis remains understudied. Through a meta-analysis of gene expression datasets followed by a mini-screen, we identified Plant Homeodomain Finger protein 8 (PHF8), a histone demethylase of the Jumonji C protein family, as a previously unidentified prometastatic gene in melanoma. Loss- and gain-of-function approaches demonstrate that PHF8 promotes cell invasion without affecting proliferation in vitro and increases dissemination but not subcutaneous tumor growth in vivo, thus supporting its specific contribution to the acquisition of metastatic potential. PHF8 requires its histone demethylase activity to enhance melanoma cell invasion. Transcriptomic and epigenomic analyses revealed that PHF8 orchestrates a molecular program that directly controls the TGFβ signaling pathway and, as a consequence, melanoma invasion and metastasis. Our findings bring a mechanistic understanding of epigenetic regulation of metastatic fitness in cancer, which may pave the way for improved therapeutic interventions.

## Teaser

PHF8 is up-regulated in metastatic melanoma and promotes invasion via transcriptional activation of TGFβ pathway genes.

## INTRODUCTION

Although metastasis accounts for more than 80% of deaths linked to solid malignancies (*1*), it is an inefficient process, as several steps hinder its progression. Tumor cells need to detach from their primary site, invade the nearby tissue, intravasate, endure circulatory stress, extravasate, and co-opt the host microenvironment to eventually establish a secondary growth (*2*). Throughout this process, cancer cells must select for genetic and epigenetic traits that are advantageous for their dissemination and adaptation to distal host organs.

The Cancer Genome Atlas (TCGA) has generated extensive genomic data across 33 cancer types, resulting in the identification of 299 driver genes for various cancer types (*3*). Particularly in melanoma, TCGA and other large sequencing efforts in the past decade have revealed genetically disrupted signaling pathways that contribute to pathogenesis, including gain-of-function mutations or amplification of proto-oncogenes (i.e., *BRAF*, *NRAS*, *KIT*, *MITF*, *CDK4*, and *MDM2*) and loss-of-function mutations or deletion of tumor suppressor genes (i.e., *PTEN*, *TP53*, *CDNK2A*, and *RB1*) (*4*). While these genetic alterations play a major role in melanoma initiation or maintenance, none fully explain metastatic behavior or the starkly different outcomes of patients initially diagnosed with primary melanoma. Recent literature supports the importance of epigenetic changes acquired by melanoma cells that lead to changes in their transcriptional output, which, in turn, increase their fitness and metastatic potential (*5*, *6*). Because malignant cells with the same genetic features can elicit diverse epigenetic programs to adapt to different microenvironments, we posit that epigenetic regulators might be critical drivers of metastasis.

Here, we mined publicly available gene expression datasets of human melanoma samples (*7*–*10*) to select a stringent subset of epigenetic regulators consistently up-regulated in metastasis as compared to primary tumors. These genes could be markers or mediators of melanoma metastasis and potentially “druggable” targets. Of the list of candidates, we focused on PHF8, a Jumonji C (JmjC) domain–containing protein that erases repressive histone marks including H4 Lysine 20 monomethyl (H4K20me1) and H3 Lysine 9 monomethyl (H3K9me1) (*11*). PHF8 is a histone demethylase that preferentially localizes at promoters and participates in cell cycle regulation by removing H4K20me1 from the promoters of E2F Transcription Factor 1 (E2F1)-regulated genes (*12*). We show that PHF8 is up-regulated in metastatic samples compared to primary melanomas and nevi from an independent patient cohort and regulates invasive and metastatic potential through a mechanism dependent on its histone demethylase function. Moreover, we demonstrate that PHF8 directly controls the transcription of invasion and metastasis-related signatures, particularly the Transforming Growth Factor–β (TGFβ) pathway, which is required for the proinvasive role of PHF8.

## RESULTS

### An unbiased approach identifies epigenetic regulators involved in melanoma maintenance or progression

**Fig. 1.**
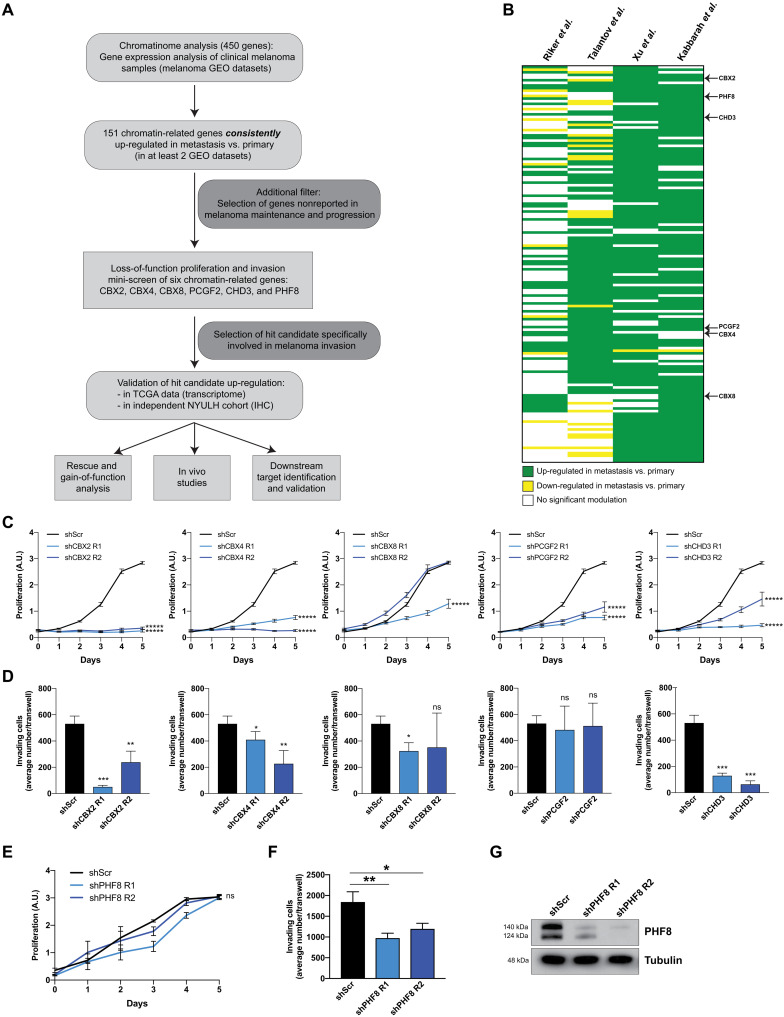
Loss-of-function targeted mini-screen identifies a role for PHF8 in melanoma invasion. (**A**) Schematic illustration of the strategy to identify chromatin-related genes specifically involved in melanoma metastasis and their downstream targets. (**B**) Data mining of four human gene expression datasets [Talantov *et al.* (GSE3189), Riker *et al.* (GSE7553), Xu *et al.* (GSE8401) and Kabbarah *et al.* (GSE46517)] shows a significant up-regulation of 151 chromatin-related genes in metastatic versus primary melanoma tumors in at least two of four datasets. Six genes were selected for a loss-of-function proliferation and invasion mini-screen. (**C**) Proliferation and (**D**) invasion assays were performed in SKMEL-147 cells transduced with two different shRNAs per chromatin-related genes (CBX2/4/8, PCGF2, and CHD3); *P < 0.05, **P < 0.01, ***P < 0.001 and *****P < 0.00001. (**E**) PHF8 knockdown did not impair SKMEL-147 proliferation (shPHF8 R1 versus shScr, *P* = 0.22; shPHF8 R2 versus shScr, *P* = 0.49) (**F**) but inhibited invasion (shPHF8 R1 versus shScr, *P* = 0.005; shPHF8 R2 versus shScr, *P* = 0.01). (**G**) Efficient PHF8 knockdown was assessed by Western blot. Error bars indicate average ± SD. ns, not significant; A.U., arbitrary units.

We sought to discover chromatin-related genes, histone-modifying enzymes, and transcription factors that potentially contribute to melanoma maintenance and/or progression. We performed a meta-analysis of four published human Affymetrix expression datasets (*7*–*10*) to identify chromatin-related genes consistently up-regulated in metastatic versus primary samples (Fig. 1A). We selected 151 chromatin-related genes that were up-regulated in metastatic versus primary tissues in at least two of the four datasets analyzed, to limit the possibility of identifying false positives (table S1). After excluding chromatin-related genes previously described as oncogenes or prometastatic genes, we chose six genes for a loss-of-function proliferation and invasion screens: chromobox 2 (CBX2), CBX4, CBX8, polycomb group finger ring 2 (PCGF2), chromodomain helicase DNA binding protein 3 (CHD3), and PHF8 (Fig. 1B and table S2). Direct roles for these chromatin-related genes in transformation or melanoma progression have not been reported. Knockdown of five of the six selected genes using two different short hairpin RNAs (shRNAs) significantly impaired proliferation of SKMEL-147 cells (Fig. 1C), with the exception of shCBX8 R2 that did not affect proliferation but proved to be inefficient for CBX8 silencing (fig. S1C). Notably, CBX2 knockdown does not only impair proliferation but also leads to apoptosis as shown by caspase-3 cleavage (fig. S1A). Invasion assays revealed decreased invasive potential upon silencing of all genes, with the exception of PCGF2 (Fig. 1D). In contrast to the other five candidates, PHF8 knockdown, which silenced two different transcript variants (Fig. 1G), did not affect proliferation (Fig. 1E) yet significantly reduced invasion in vitro (Fig. 1F). To decipher mechanisms directly affecting metastasis and patient outcomes, we selected a candidate hit that specifically altered melanoma invasive potential without affecting cell proliferation. Therefore, while all tested candidates merit further characterization of their role in melanoma maintenance and progression, we elected to further examine the role of PHF8 in melanoma invasion and metastasis.

### PHF8 consistently regulates melanoma invasion

**Fig. 2.**
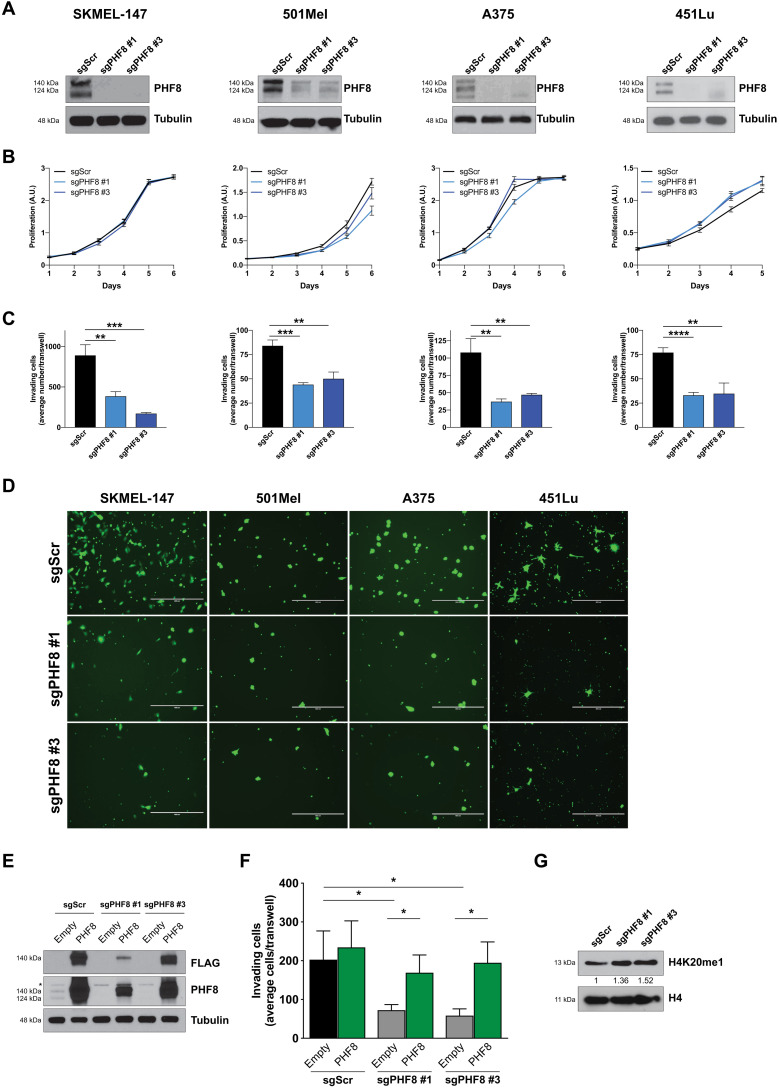
PHF8 gene silencing inhibits invasion but not proliferation in vitro. (**A**) Efficient PHF8 knockout using two different sgRNAs in four different melanoma cell lines was determined by Western blot. The membrane was reblotted with tubulin antibody for loading control. (**B**) Proliferation curves of cells seeded 3 days after infection with sgRNA-carrying lentiviral particles targeting PHF8. (**C**) Invasion assay of cells seeded 3 days after infection with sgRNA-containing lentiviral particles, using Fluoroblok transwell chambers coated with Matrigel. PHF8 knockout significantly inhibits melanoma invasion in SKMEL-147 (sgPHF8 #1 versus sgScr, *P* = 0.003; sgPHF8 #3 versus sgScr, *P* = 0.0007), 501Mel (sgPHF8 #1 versus sgScr, *P* = 0.0003; sgPHF8 #3 versus sgScr, *P* = 0.002), A375 (sgPHF8 #1 versus sgScr, *P* = 0.003; sgPHF8 #3 versus sgScr, *P* = 0.006), and 451Lu cells (sgPHF8 #1 versus sgScr, *P* = 0.0001; sgPHF8 #3 versus sgScr, *P* = 0.003). (**D**) Representative pictures of the Fluoroblok transwell inserts stained with Calcein AM to quantify invading cells in (C). Scale bars, 400 μm. (**E**) PHF8 expression was restored in SKMEL-147 cells upon infection of cells previously transduced with sgScr, sgPHF8 #1, or sgPHF8 #3 with Empty or FLAG-HA-PHF8 overexpressing lentiviral particles. Membrane blotting with anti-PHF8 shows both sgRNA efficiency and PHF8 overexpression. Exogenous PHF8 expression was assessed by Western blot of the FLAG tag. (**F**) Invasion assay demonstrates that ectopic PHF8 expression restores the invasive potential of PHF8 knockout cells (sgPHF8 #1 versus sgScr, *P* = 0.048; sgPHF8 #3 versus sgScr, *P* = 0.039; sgPHF8 #1 + PHF8 WT versus sgPHF8 #1 + Empty, *P* = 0.026; sgPHF8 #3 + PHF8 WT versus sgPHF8 #3 + Empty, *P* = 0.014). Error bars indicate average ± SD. Representative data from three independent experiments conducted in each cell line are shown. (**G**) SKMEL-147 cells transduced as indicated were analyzed for H4K20me1 global levels by immunoblotting. H4 blot serves as a control for protein loading. Band intensity relative to sgScr condition is shown.

With the aim of ruling out off-target effects, we used orthogonal methods to knock out PHF8 and assess how general the proinvasive effect of PHF8 is across multiple melanoma cell lines. The loss of invasive capacity observed upon PHF8 knockdown in SKMEL-147 melanoma cells was consistently reproduced using the CRISPR-Cas9 system and two efficient small guide RNAs (sgRNAs) targeting PHF8 (sgPHF8 #1 and sgPHF8 #3) in four melanoma cell lines (SKMEL-147, 501Mel, A375, and 451Lu) (Fig. 2, A to D). PHF8 knockout did not affect proliferation (Fig. 2B) but significantly inhibited invasion (Fig. 2, C and D). The four cell lines used differ in *NRAS* and *BRAF* mutational status, suggesting that PHF8 proinvasive phenotype is not restricted to a particular genetic background. To further confirm the specificity of the observed effects, SKMEL-147 cells stably transduced with PHF8 sgRNAs or scrambled sgRNA (sgScr) were infected with FLAG-hemagglutinin (HA)–tagged PHF8 overexpressing lentiviruses. PHF8 ectopic expression, confirmed by FLAG and PHF8 immunoblots (Fig. 2E), was able to rescue the defect in invasion caused by PHF8 knockout (Fig. 2F). PHF8 loss led to increased H4K20me1 deposition relative to control, sgScr-transduced SKMEL-147 cells (Fig. 2G), as previously demonstrated by Liu *et al.* (*12*).

### PHF8 is up-regulated in metastatic melanoma patient samples

**Fig. 3.**
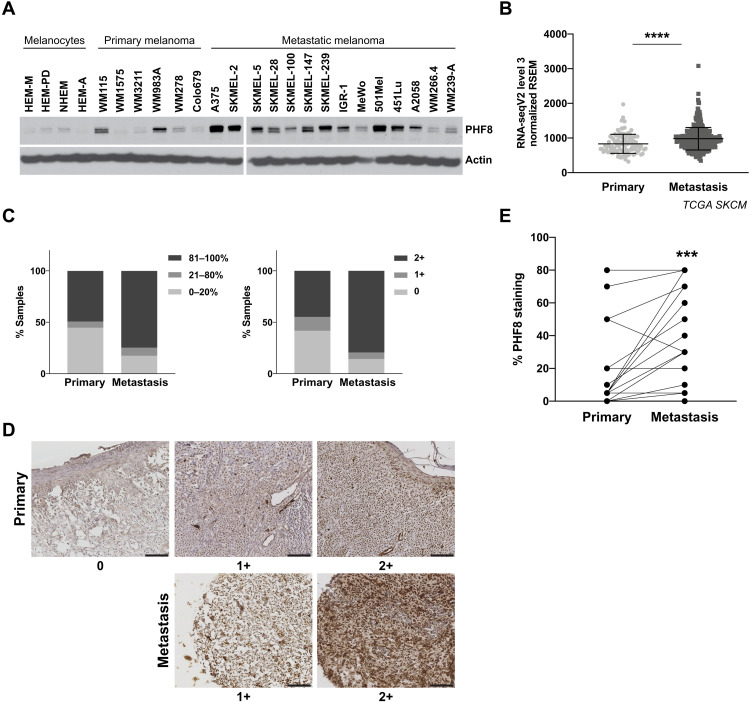
PHF8 expression is associated with metastasis in melanoma patient samples. (**A**) PHF8 protein levels in cultured human epidermal melanocytes (neonatal, HEM-M and HEM-D; adult NHEM and HEM-A), primary and metastatic human melanoma cell lines, detected by Western blot. Actin blotting was used as loading control. (**B**) Dot plot of *PHF8* expression in primary (*n* = 103) and metastatic (*n* = 369) samples of TCGA, showing a significant up-regulation of *PHF8* mRNA expression in metastatic versus primary melanoma tumors (*P* < 0.0001). (**C**) Immunochemistry staining of PHF8 in melanoma tumor samples from an independent NYU Langone Health cohort (primary *n* = 67 and metastasis *n* = 46; *P* = 0.002 for PHF8 high tumor percentage staining/intensity in metastatic versus primary; Mann-Whitney test). Staining was scored according to the intensity (0 to 1+ to 2+) and distribution (percentage of tumor with positive staining). (**D**) Representative images of different staining intensities of PHF8 immunostaining are shown for primary and metastatic patients’ samples. Scale bar, 100 μm. (**E**) PHF8 expression in 22 metastatic melanoma cases and their patient-matched primary tumors, assessed by PHF8 immunohistochemistry (*P* = 0.0004, two-tailed paired *t* test).

We analyzed PHF8 protein expression in a panel of human cultured melanocytes (*n* = 4), primary (*n* = 7) and metastatic (*n* = 11) melanoma cell lines. PHF8 expression was low or barely detectable in melanocytes derived from perinatal and adult skin. Primary cell lines displayed variable levels, whereas most metastatic melanoma cell lines expressed high PHF8 levels (Fig. 3A). Similar differences were observed at transcript levels by *PHF8* quantitative real-time polymerase chain reaction (qRT-PCR) (fig. S2). *PHF8* mRNA was found significantly up-regulated in metastatic relative to primary patient samples from TCGA data (Fig. 3B) (*13*), as well as in two of the Affymetrix datasets used in our meta-analysis (Fig. 1B): Xu *et al.* (GSE8401) and Kabbarah *et al.* (GSE46517). To further validate PHF8 overexpression in metastatic melanoma, we performed PHF8 immunohistochemistry on an independent cohort of primary (*n* = 67) and metastatic (*n* = 46) melanoma patient samples obtained from the New York University (NYU) Langone Health Melanoma Program. Consistent with our findings in TCGA and in melanoma cell lines, we observed that while primary samples were similarly split between no/low and high PHF8 expression, most metastatic samples expressed high PHF8 levels, both in percentage of positive cells and staining intensity (Fig. 3C). We therefore establish that elevated *PHF8* transcriptional levels in metastatic samples relative to primary samples—observed in public gene expression datasets—are consistent with higher PHF8 protein levels. Representative images show different nuclear PHF8 staining intensities in primary and metastatic samples (Fig. 3D). In addition, in a subset of 22 patient-matched melanoma samples, we found a statistically significant increase in PHF8 expression from primary to metastasis (Fig. 3E). These findings link PHF8 expression to melanoma metastasis and disease progression.

### PHF8 promotes metastasis in vivo

**Fig. 4.**
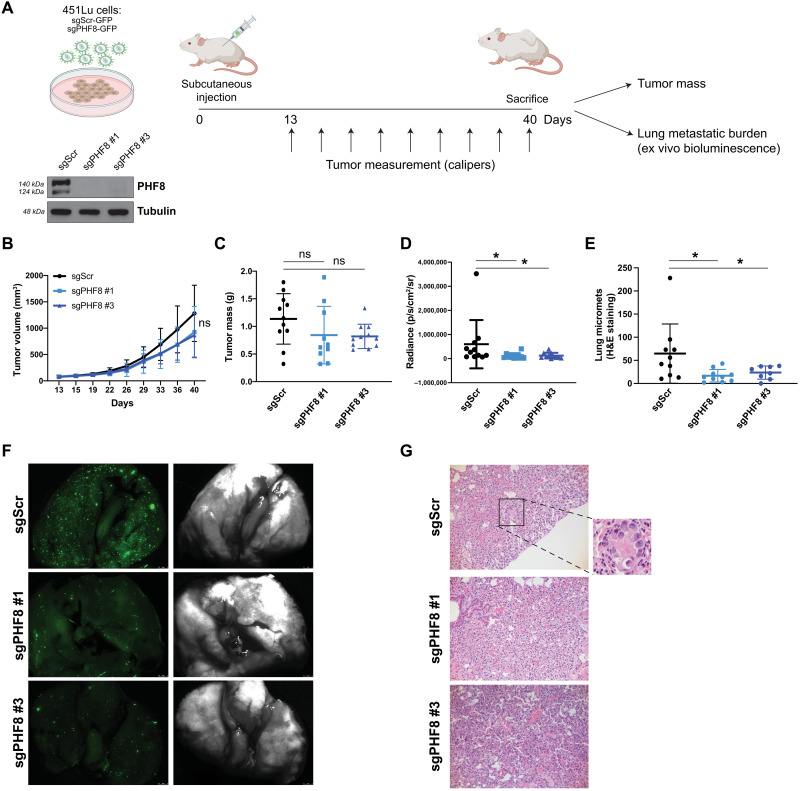
PHF8 knockout in melanoma cells inhibits metastasis in vivo. (**A**) Schematic representation of the in vivo metastasis assay: 451Lu cells stably expressing luciferase for in vivo bioluminescence imaging and Cas9 were transduced with control (sgScr) or PHF8 sgRNA (sgPHF8#1 and sgPHF8 #3) lentiviral particles carrying a green fluorescent protein (GFP) reporter and subcutaneously injected into NOD/Scid/IL2γRnull female mice. (**B**) PHF8 knockout does not affect tumor growth (sgPHF8 #1 versus sgScr, *P* = 0.12; sgPHF8 #3 versus sgScr, *P* = 0.06) (**C**) or tumor mass (sgPHF8 #1 versus sgScr, *P* = 0.17; sgPHF8 #3 versus sgScr, *P* = 0.06) at termination of the experiment. (**D**) PHF8 knockout inhibits melanoma metastasis to the lungs. Tumor burden was measured by bioluminescent imaging (IVIS) of dissected lungs 5 min after mouse euthanasia. (sgPHF8 #1 versus sgScr, *P* = 0.029; sgPHF8 #3 versus sgScr, *P* = 0.023). Error bars indicate mean ± SD. (**E**) Lung sections were sliced at three different levels, followed by H&E staining, and the number of micrometastatic foci was counted by a pathologist (sgPHF8 #1 versus sgScr, *P* = 0.015; sgPHF8 #3 versus sgScr, *P* = 0.045). (**F**) Representative images of lungs acquired with a dissecting scope before fixation allow visualizing GFP-expressing melanoma metastatic foci. (**G**) Hematoxylin and eosin (H&E)–stained sections of lungs resected at termination. Inset displays a metastatic lesion in the sgScr group.

To investigate the effect of PHF8 knockout on the metastatic capacity of melanoma cells in vivo, 451Lu cells transduced with a luciferase-expressing construct, Cas9 and sgPHF8 #1, sgPHF8 #3, or sgScr were injected subcutaneously in the flanks of immune-compromised mice. Once palpable, tumor growth was regularly measured by caliper (Fig. 4A). Although PHF8 knockout did not affect primary tumor growth (Fig. 4B) or tumor mass at termination of the experiment (40 days) (Fig. 4C), it led to a significant decrease in lung metastasis burden measured by ex vivo bioluminescence (Fig. 4D), which corresponded to a reduced number of metastatic foci (Fig. 4, E to G). We conclude that PHF8 enhances melanoma metastatic progression without affecting primary tumor growth.

### PHF8 regulates melanoma invasive potential through its histone demethylase function

**Fig. 5.**
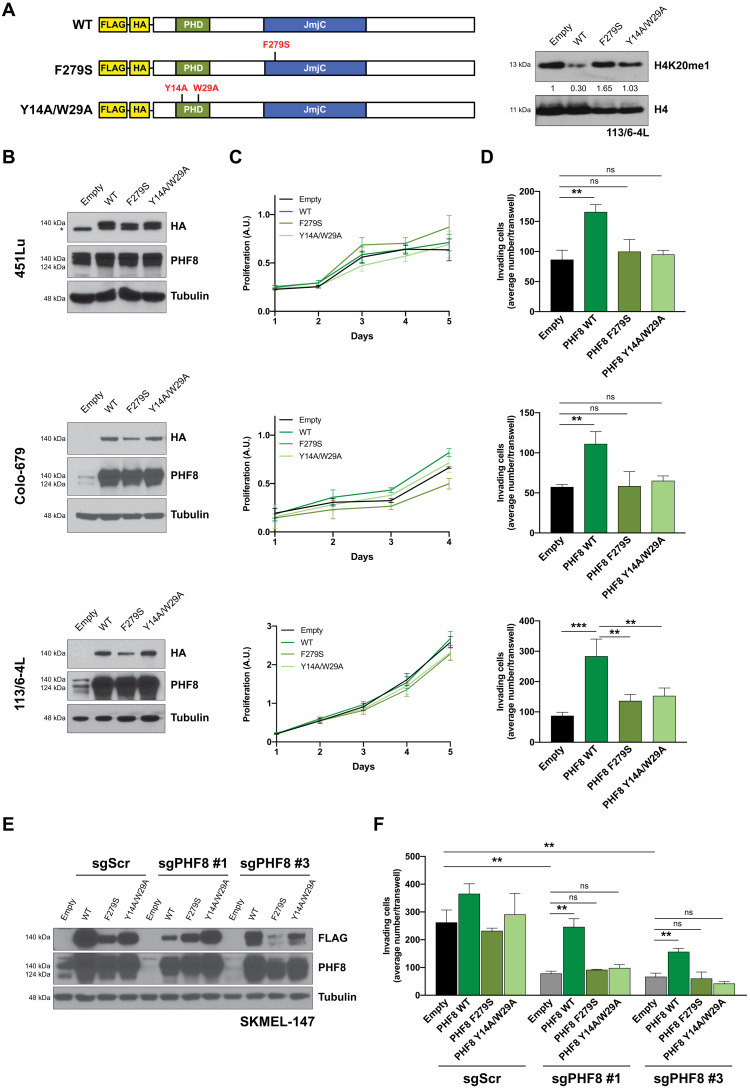
PHF8 histone demethylase activity is required for its proinvasive function. (**A**) Left: Lentiviral constructs encoding for FLAG-HA tagged PHF8 WT, F279S, and Y14A/W29A. Right: Immunoblotting of H4K20me1 in 113/6-4L overexpressing PHF8 WT or PHF8 mutants. H4 Western blot serves as loading control. Band intensity relative to Empty condition is shown. (**B**) Efficient overexpression of PHF8 WT and mutant constructs in three cell lines was determined by Western blot using HA and PHF8 antibodies. (**C**) Proliferation and (**D**) Invasion assays were performed in 451Lu (top: PHF8 WT versus Empty, *P* = 0.002; PHF8 F279S versus Empty, *P* = 0.42; PHF8 Y14A/W29A versus Empty, *P* = 0.43), Colo-679 (middle: PHF8 WT versus Empty, *P* = 0.004; PHF8 F279S versus Empty, *P* = 0.91; PHF8 Y14A/W29A versus Empty, *P* = 0.13), and 113/6-4L cells (bottom: PHF8 WT versus Empty, *P* = 0.0004; PHF8 F279S versus PHF8 WT, *P* = 0.002; PHF8 Y14A/W29A versus PHF8 WT, *P* = 0.005). (**E**) Efficient overexpression of PHF8 WT and mutant constructs in SKMEL-147 cells previously transduced with sgScr or sgPHF8 #3 was determined by Western blot. (**F**) Invasion assay shows that ectopic PHF8 WT expression, but not the mutant constructs, restores the invasive potential of PHF8 knockout SKMEL-147 cells (sgPHF8 #1 + Empty versus sgScr + Empty, *P* = 0.005; sgPHF8 #1 + PHF8 WT versus sgPHF8 #1 + Empty, *P* = 0.002; sgPHF8 #1 + PHF8 F279S versus sgPHF8 #1 + Empty, *P* = 0.14; sgPHF8 #1 + PHF8 Y14A/W29A versus sgPHF8 #1 + Empty, *P* = 0.089; sgPHF8 #3 + Empty versus sgScr + Empty, *P* = 0.004; sgPHF8 #3 + PHF8 WT versus sgPHF8 #3 + Empty, *P* = 0.004; sgPHF8 #3 + PHF8 F279S versus sgPHF8 #3 + Empty, *P* = 0.69; sgPHF8 #3 + PHF8 Y14A/W29A versus sgPHF8 #3 + Empty, *P* = 0.098). Error bars indicate average ± SD. Representative data of three independent experiments conducted are shown. Western blot membranes were reblotted with tubulin antibody for loading control.

Elevated *PHF8* mRNA in metastatic samples is consistent with higher nuclear protein expression by immunohistochemistry (Fig. 3, C to E), suggesting that its role as histone demethylase and transcriptional activator could contribute to its proinvasive effect. We addressed whether PHF8 demethylase activity is required for its role in melanoma invasion. We used two different mutant constructs that impair the PHF8 demethylase activity in different ways (*11*). PHF8 F279S harbors a single point mutation in the JmjC domain that impairs its catalytic activity, while the PHF8 Y14A/W29A construct contains two mutations in the PHD domain (Fig. 5A). PHF8 binds to H3K4me3, a histone mark of active promoters, via its Plant Homeodomain (PHD) domain, allowing the linker region between the PHD and JmjC domain to adopt a bent conformation and interact with and demethylate H3K9me1/me2 (*14*). Therefore, the latter mutant construct also impairs the demethylase activity by preventing PHF8 recruitment to transcription start sites (TSSs). The overexpression of wild-type (WT) PHF8, but not of its mutant forms, decreases H4K20me1 levels (Fig. 5A, right). We stably transduced 451Lu, Colo-679, and 113/6-4L (*15*) melanoma cell lines with Empty, PHF8 WT, PHF8 F279S, or PHF8 Y14A/W29A-carrying lentiviral particles (Fig. 5B). While none of the transduced PHF8 WT or mutant constructs significantly affected proliferation (Fig. 5C), we observed that PHF8 WT overexpression significantly increases invasion in all three cell lines (Fig. 5D). These data support that PHF8 overexpression is sufficient to increase melanoma cell invasion. However, both mutant forms failed to promote invasion, demonstrating that the demethylase activity of PHF8 is required for its role in invasion (Fig. 5D). Accordingly, only PHF8 WT, and not the mutants lacking demethylase activity, was able to rescue the defect in invasion observed in PHF8 knockout melanoma cells (Fig. 5, E and F).

### PHF8 controls the transcription of invasion and metastasis-related signatures

**Fig. 6.**
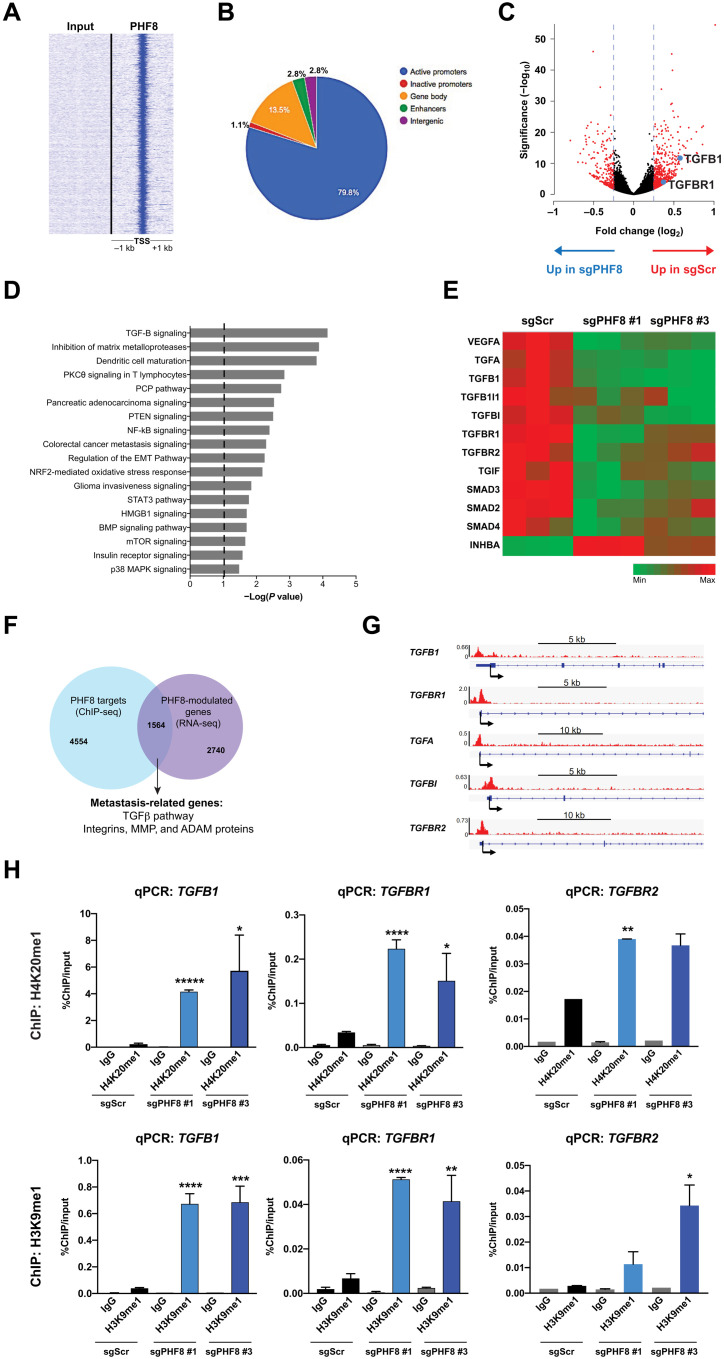
PHF8 directly modulates metastasis-related genes. (**A**) Heatmap representation of ChIP-seq binding for PHF8 peaks on 1-kb flanked TSS regions, using seqMiner. Input is shown as a negative control for enrichment. (**B**) Genome-wide distribution of PHF8 on active (H3K9me3)/inactive (H3K9me3/H3K27me3) promoters, gene bodies (excluding flanked TSS regions), enhancers (H3K27Ac/H3K4me1), and intergenic regions (excluding all of the above) by overlapping PHF8 ChIP-seq with H3K9me3, H3K4me1, H3K27Ac, and H3K27me3 ChIP-seq performed in SKMEL-147 cells (*60*). (**C**) RNA-seq was performed in SKMEL-147 transduced with sgScr or PHF8 sgRNAs. Scatterplots of gene expression versus fold change (log_2_) expression between sgPHF8- and sgScr-transduced cells. Genes significantly modulated in sgPHF8 versus sgScr cells are depicted in red. (**D**) Top biological pathways modulated by PHF8 are identified using the Ingenuity Pathway. The vertical line represents the significance threshold (*P* value of 0.05). PCP, phencyclidine; NF-κB, nuclear factor κB; BMP, bone morphogenetic protein; mTOR, mammalian target of rapamycin; PTEN, phosphatase and tensin homolog; HMGB1, high mobility group box 1. (**E**) Heatmap of genes involved in the TGFβ signaling pathway and significantly regulated by PHF8 based on RNA-seq data as described in (C) and (D). Rows represent normalized counts per gene. VEGFA, vascular endothelial growth factor A; TGIF, TGFB induced factor homeobox 1; INHBA, inhibin subunit beta A. (**F**) Venn diagram overlapping ChIP-seq targets and PHF8-modulated genes (RNA-seq) identifies direct PHF8 targets in melanoma, encompassing metastasis-related genes. (**G**) ChIP-seq tracks of PHF8 binding to the TSS of direct targets that are TGFβ genes. (**H**) H4K20me1 and H3K9me1 ChIP experiments followed by qPCR of *TGFB1*, *TGFBR1*, and *TGFBR2* regions bound by PHF8 were performed in SKMEL-147 transduced with sgPHF8 #1, sgPHF8 #3, or scrambled control (sgScr): H4K20me1 ChIP *TGFB1* qPCR (sgPHF8 #1 versus sgScr, *P* = 0.000003; sgPHF8 #3 versus sgScr, *P* = 0.030), H4K20me1 ChIP *TGFBR1* qPCR (sgPHF8 #1 versus sgScr, *P* = 0.000098; sgPHF8 #3 versus sgScr, *P* = 0.032), H4K20me1 ChIP *TGFBR2* qPCR (sgPHF8 #1 versus sgScr, *P* = 0.002; sgPHF8 #3 versus sgScr, *P* = 0.16), H3K9me1 ChIP *TGFB1* qPCR (sgPHF8 #1 versus sgScr, *P* = 0.0001; sgPHF8 #3 versus sgScr, *P* = 0.0007), H3K9me1 ChIP *TGFBR1* qPCR (sgPHF8 #1 versus sgScr, *P* = 0.0001; sgPHF8 #3 versus sgScr, *P* = 0.007), and H3K9me1 ChIP *TGFBR2* qPCR (sgPHF8 #1 versus sgScr, *P* = 0.1; sgPHF8 #3 versus sgScr, *P* = 0.03). IgG, immunoglobulin G.

To investigate how PHF8 contributes to the metastatic process, we further delved into the transcriptional changes triggered by PHF8 modulation in melanoma cells. Chromatin immunoprecipitation sequencing (ChIP-seq) analyses reveal that PHF8 binds mostly active promoters (Fig. 6, A and B), which concurs with previous studies describing its function as a transcriptional activator (*12*). However, although we were able to recapitulate the antiproliferative effects and *E2F1* down-regulation previously observed in HeLa cells upon PHF8 depletion (fig. S3, A to F) (*12*), we found that, unlike *TGFB1* promoter, the *E2F1* promoter was not significantly bound by PHF8 in SKMEL-147 cells (fig. S3G). In addition, we consistently show using multiple cell lines that *E2F1* is not a transcriptional target of PHF8 in melanoma (fig. S3, H and I). To better understand the molecular mechanisms underlying PHF8 regulation of invasion and metastasis, we performed RNA sequencing (RNA-seq) to compare the transcriptome of sgPHF8 (using two different sgRNAs) versus sgScr-transduced cell lines. A significant number of genes (4304 genes) are differentially regulated in sgPHF8 relative to sgScr-transduced cells [*P* < 0.05; false discovery rate (FDR) ≤ 0.15] (Fig. 6C). Ingenuity Pathway Analysis was performed on a smaller list of 2573 genes (*P* < 0.01; FDR < 0.05) to which an additional cutoff of changes in expression was applied (−0.3 < log_2_ fold change < +0.3). This analysis revealed that PHF8 controls the transcription of multiple invasion and metastasis-related genes, such as integrins, matrix metalloproteinases (MMPs), and A Disintegrin and Metalloproteinase (ADAM) proteins, as well as TGFβ signaling, the most significantly modulated pathway at the transcriptional level (Fig. 6D). Several ligands, receptors, and transcription factors involved in the TGFβ signaling pathway are positively regulated by PHF8 (Fig. 6E). The overlap of PHF8 targets identified by ChIP-seq (6118 significant peaks) with the 4304 genes transcriptionally modulated by PHF8 deletion revealed 1564 genes, the expression of which is directly regulated by PHF8 binding. This category comprises MMPs, integrins, and ADAM proteins, as well as genes of the canonical TGFβ signaling pathway (Fig. 6F), which are directly bound by PHF8 at their TSS (Fig. 6G). As another readout of PHF8 loss-of-function, we examined the deposition of its major substrate and repressive histone marks, H3K9me1 and H4K20me1, at promoter regions of PHF8 direct targets, *TGFB1*, *TGFBR1*, and *TGFBR2*. ChIP-qPCR data indicate that those histone marks are enriched in sgPHF8-transduced cells as compared to their scrambled control at the TSS regions of *TGFB1*, *TGFBR1*, and *TGFBR2* that are occupied by PHF8 (Fig 6, G and H). Moreover, overexpression of PHF8 WT, but not of its mutant forms, suppresses H4K20me1 and H3K9me1 deposition at the TSS regions of *TGFB1* and *TGFBR1* (fig. S4). Together, our data demonstrate that PHF8 is a direct transcriptional activator of prometastatic genes, notably several genes of the TGFβ pathway, via demethylation of its substrates, the repressive histone marks H3K9me1 and H4K20me1.

### The TGFβ pathway is directly regulated by PHF8 and required for its proinvasive role

**Fig. 7.**
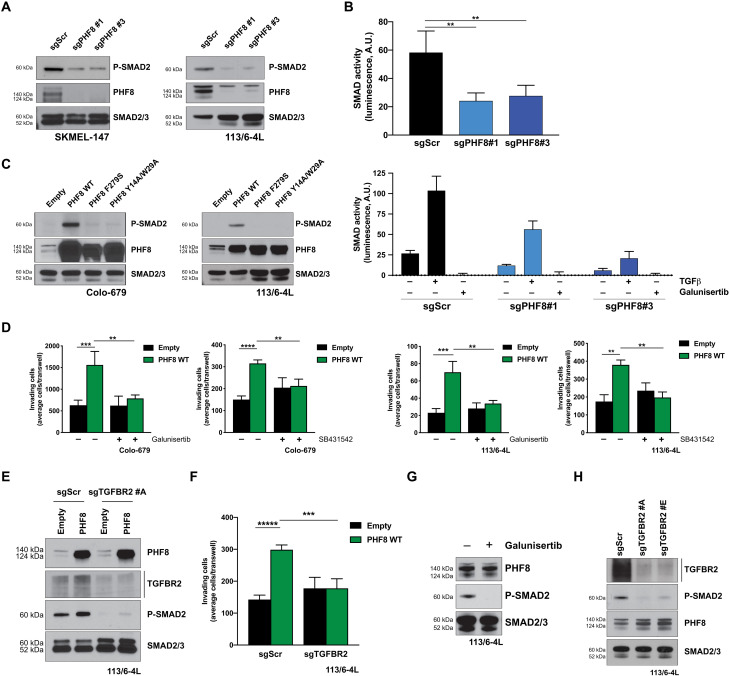
PHF8 directly targets TGFβ pathway activation, which is required for melanoma invasion. (**A**) P-SMAD2 Western blot shows down-regulation of TGFβ signaling upon PHF8 knockout in SKMEL-147 and 113/6-4L cells. (**B**) SKMEL-147 cells were transduced as indicated, along with an SBE luciferase reporter lentivirus. Top: Luciferase assay shows significant reduction in SMAD activity upon PHF8 knockout (sgPHF8 #1 versus sgScr, *P* = 0.002; sgPHF8 #3 versus sgScr, *P* = 0.002). Bottom: SKMEL-147 cells transduced as in the left panel were serum-deprived overnight before treatment with TGFβ (10 ng/ml) or galunisertib (10 μM) for 12 hours, followed by measurement of SMAD activity by luciferase assay. (**C**) P-SMAD2 Western blot shows induction of TGFβ signaling in Colo-679 and 113/6-4L cells overexpressing PHF8 WT, but not PHF8 mutant forms, as compared to their Empty-transduced control. (**D**) Colo-679 and 113/6-4L cells transduced with PHF8 WT or Empty lentiviral particles were treated with TGFβ inhibitors galunisertib or SB431542 (10 μM) for 24 hours before invasion assays were performed. [Colo-679: PHF8 WT versus Empty (without galunisertib), *P* = 0.001; PHF8 WT + galunisertib versus PHF8 WT (without galunisertib), *P* = 0.003; PHF8 WT versus Empty (without SB431542), *P* = 0.00001; PHF8 WT + SB431542 versus PHF8 WT (without SB431542), *P* = 0.002] and [113/6-4L: PHF8 WT versus Empty (without galunisertib), *P* = 0.0005; PHF8 WT + galunisertib versus PHF8 WT (without galunisertib), *P* = 0.001; PHF8 WT versus Empty (without SB431542), *P* = 0.0017; PHF8 WT + SB431542 versus PHF8 WT (without SB431542), *P* = 0.0017]. (**E**) Western blot of cell lysates from 113/6-4L cells transduced with Cas9-KRAB and Empty or PHF8 overexpressing lentiviral particles, followed by sgTGFBR2 #A or sgScr transduction. TGFBR2 silencing efficiently suppresses P-SMAD2 induction by PHF8. (**F**) The invasive potential of cells shown in (E) was assessed. PHF8-induced invasion is rescued by the inhibition of the TGFβ pathway via TGFBR2 depletion (PHF8 WT + sgScr versus Empty + sgScr, *P* = 0.000006; PHF8 WT + sgTGFBR2 versus PHF8 WT + sgScr, *P* = 0.0004). (**G**) Western blot analyses in 113/6-4L cells treated with galunisertib (**H**) or transduced with sgRNAs targeting TGFBR2 show that inhibition of the TGFβ pathway does not reduce PHF8 endogenous levels. Error bars indicate average ± SD. Representative data of three independent experiments are shown.

**Fig. 8.**
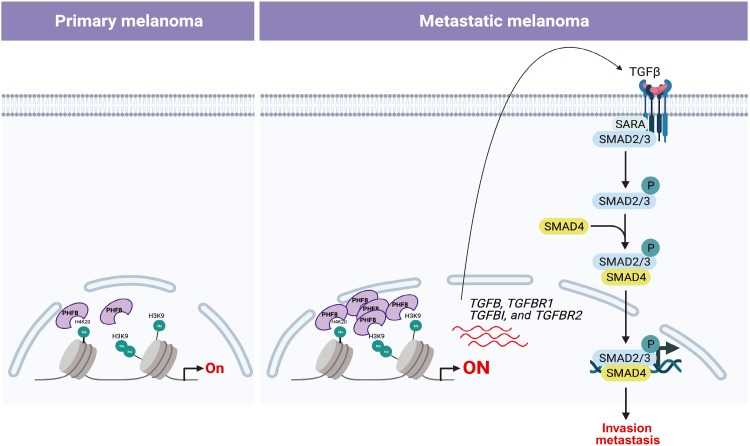
Schematic of PHF8 molecular mechanism during melanoma progression.

Further proving the regulation of TGFB1 by PHF8, we show reduced secreted TGFβ1 in media from PHF8-depleted SKMEL-147 cells (fig. S5A). Consequently, PHF8 silencing leads to up-regulation of *ID1* and *ID2*, which are suppressed by the TGFβ pathway in melanoma (fig. S5B). As shown in Fig. 6E, PHF8 knockout down-regulates *TGFB1*, *TGFBR1*, and *TGFBI*, a downstream signaling target of the TGFβ signaling cascade (fig. S5C). Therefore, we postulated that PHF8 positively regulates TGFβ signaling. The canonical TGFβ signaling cascade is initiated when TGFβ ligands (i.e., TGFB1) bind to the type II transmembrane receptor serine/threonine kinase TGFBR2, which, in turn, assembles with, phosphorylates, and activates the type I receptor TGFBR1. Activated TGFBR1 phosphorylates the downstream effectors SMAD2 and SMAD3, which then associate with SMAD4. The formed complex accumulates in the nucleus where it regulates the transcription of various target genes (*16*). We found that basal levels of P-SMAD2 (Ser^465/467^), a marker of TGFβ signaling, are notably down-regulated in sgPHF8-transduced SKMEL-147 and 113/6-4L cells as compared to their sgScr control (Fig. 7A). Consistent with these findings, PHF8 loss-of-function reduced SMAD transcriptional activity, as measured by a luciferase reporter assay (Fig. 7B, top). Changes in SMAD activity in the presence of TGFβ or galunisertib support the specificity of the reporter assay (Fig. 7B, bottom). Conversely, PHF8 overexpression in Colo-679 and 113/6-4L cells resulted in increased SMAD2 phosphorylation relative to empty vector–transduced control cells (Fig. 7C). PHF8 induces P-SMAD2 in a histone demethylase–dependent manner, because PHF8 mutants are unable to up-regulate P-SMAD2 (Fig. 7C). Furthermore, we established that TGFβ signaling is required for PHF8 proinvasive phenotype, because the increase in invasion observed upon PHF8 overexpression in Colo-679 and 113/6-4L cells (Fig. 7D) can be significantly reversed by pre-treatment with TGFβ receptor inhibitors galunisertib and SB431542 (Fig. 7D) or genetic deletion of TGFBR2 (Fig. 7F). We further confirm that PHF8 is an upstream regulator of TGFβ signaling, because efficient pharmacological (Fig. 7G) or genetic inhibition of this pathway does not reduce PHF8 expression (Fig. 7H). Overall, these data demonstrate that PHF8 directly orchestrates a transcriptional proinvasive program that comprises several components of the TGFβ pathway, activates TGFβ signaling, and promotes melanoma invasion and metastasis (Fig. 8). In agreement with our proposed model, *PHF8* levels positively correlate with the expression of its transcriptional targets (e.g., *SMAD3* and *SMAD4*; Fig. 6E) across multiple transcriptomic datasets [TCGA Skin Cutaneous Melanoma (SKCM) (*13*), Kabbarah *et al.* (*7*), and Xu *et al.* (*10*)] in which PHF8 was found up-regulated in metastasis versus primary melanoma (fig. S6).

## DISCUSSION

Melanomas are highly metastatic tumors, yet despite intensive genome sequencing efforts, no genetic alterations that explain metastatic progression and melanoma patient outcomes have been identified. Here, we investigated the ability of epigenetic regulators, which orchestrate changes in transcriptional programs, to confer metastatic fitness to malignant cells. We performed an unbiased meta-analysis of publicly available gene expression datasets to identify candidate epigenetic regulators altered in metastatic versus primary melanoma. We expected this approach would reduce false positives in candidate selection by limiting biases due to differences in sample processing, technical considerations, and cohort size and increase our chances of discovering genes truly involved in melanoma progression. All six chromatin-related genes tested in our loss-of-function mini-screen proved to be essential for melanoma proliferation and/or invasion, thus supporting the value of our strategy. CBX2, CBX4, and CBX8 are canonical components of the Polycomb repressive complex 1 (PRC1), responsible for its targeting to chromatin through physical interaction with H3K9me3 and H3K27me3 marks via their chromodomains (*17*, *18*). PCGF2 is also a component of the PRC1 core complex and maintains the transcriptional repression of genes involved in embryogenesis, cell cycle, and tumor suppression (*19*). Polycomb group proteins have been involved in a variety of biological processes, such as X-chromosome inactivation, maintenance of pluripotency, self-renewal capacity in embryonic stem cells, cell fate decisions, and developmental processes (*20*). Moreover, Polycomb genes are frequently found mutated or deregulated in cancer (*21*, *22*). CHD3 is a chromatin remodeler containing a SNF2-related helicase/adenosine triphosphatase domain and a component of the histone deacetylase complex referred to as the Mi-2/NuRD complex, which participates in the remodeling of chromatin through histone deacetylation (*23*). Each of these genes represents attractive candidates as therapeutic targets for advanced melanoma and awaits further investigation in follow-up studies. To better understand mechanisms of melanoma metastasis, we opted to focus on PHF8, the modulation of which affected invasion without altering cell proliferation.

Melanoma cells are able to switch via transcriptional reprogramming between cellular states, categorized as proliferative or invasive, both of which are marked by distinct gene expression signatures (*24*, *25*). Because individual cells have been shown to switch back and forth between those states, genetic mutations cannot underlie these cellular phenotypes (*26*, *27*). However, epigenetic factors might trigger changes in transcriptional output that ultimately increase melanoma cells aggressiveness. Hence, isolated melanoma cells expressing the H3K4 demethylase JARID1B characterize a slow-cycling tumorigenic cell population (*28*) that is associated with resistance to therapy (*29*).

Here, we identified PHF8 as a prometastatic factor. PHF8 is a histone demethylase that binds through its PHD domain to H3K4me3, an active histone mark located at TSSs, and is thus recruited and enriched at target gene promoters (*30*). Notably, PHF8 plays a role in various developmental and disease processes. Mutations in *PHF8* are associated with X-linked mental retardation and cleft lip/cleft palate (*31*), and the modulation of histone methylation by PHF8 plays a critical role in neuronal differentiation and brain and craniofacial development (*11*). PHF8 overexpression in malignant cells has been reported in several cancers, including acute lymphoblastic leukemia (*32*), breast cancer (*33*), colorectal cancer (*34*), gastric cancer (*35*, *36*), prostate cancer (*37*–*40*), and hepatocellular carcinoma (*41*). However, PHF8 occupancy and its downstream transcriptional programs in those tumor types have not been elucidated. We report that, unlike other cell types such as HeLa cells (fig. S3), PHF8 does not regulate cell cycle progression in melanoma. It is critical to gain a better understanding of the downstream mechanisms of PHF8 in various contexts, and whether modulation of the TGFβ pathway is a more general finding.

The modulation of TGFβ signaling and invasion by PHF8 offers the possibility of targeting PHF8 to inhibit this pathway. To date, while inhibitors of the JmjC demethylases have been reported, few have shown sufficient potency and selectivity toward subfamily members sharing high sequence identity, such as PHF8, PHF2, and Lysine Demethylase 7A (KDM7A or KIAA1718) (*42*). The development of novel pyridine derivatives in which the introduction of specific substituents is used to modulate the selectivity profile against histone lysine demethylases is ongoing (*43*), but their efficacy in cellular assays has not yet been reported.

We found that PHF8 epigenetically regulates TGFβ signaling through transcriptional activation of ligands and receptors of the pathway. Shao *et al.* (*44*) reported that MYC posttranscriptionally regulates PHF8 in breast cancer. In this context, MYC and TGFβ treatment cooperate to up-regulate PHF8, thus contributing to proliferation and epithelial-mesenchymal transition (EMT) through transcriptional up-regulation of *SNAI1* and *ZEB1*. They did not report TGFB1 and its receptors as direct downstream targets of PHF8, as we find in melanoma. In further contrast, our transcriptomic analyses did not reveal a modulation of EMT genes, including *ZEB1*, but rather pointed to a TGFβ pathway signature, encompassing ligands and receptors, as direct PHF8 downstream effectors. These findings highlight the pleiotropic roles of epigenetic regulators across different cellular and tumor types. Among the genes directly modulated by PHF8, we found TGFβ-induced (TGFBI) (Fig. 6, E and G), a secreted extracellular matrix component that confers high metastatic potential to melanoma cells (*45*). Cell autonomous activation of the TGFβ pathway in melanoma cell lines has been well documented (*46*). TGFβ signaling through TGFBR2 expression enhances melanoma invasion and motility, and TGFβ ligands induce their own expression through a positive amplification loop (*47*). Clinically, one study reported that TGFβ plasma levels are elevated in patients with melanoma and correlate with metastatic progression (*48*).

Unfortunately, harnessing the TGFβ pathway as a therapeutic target in cancer has long been hindered by the pleiotropic nature of its signaling effects. In early-stage tumors, TGFβ pathway activation leads to cytostatic and apoptotic tumor-suppressive responses. However, tumor cells hijack the tumor-suppressive responses to TGFB1 and convert this signal into an oncogenic factor (*49*). Intriguingly, genome-wide expression analysis of nearly 100 human melanoma cell lines demonstrated that the coordinated expression of a number of genes reminiscent of a TGFβ signature associates with a highly invasive phenotype and low proliferation rate (*27*). Therefore, the inhibition of the TGFβ signaling cascade could be effective in preventing dissemination. Several TGFβ-targeting agents, used as single agents or in combination, have been tested in clinical trials with some promising results in different cancers (*50*). TGFβ signaling can cross-talk with the mitogen-activated protein kinase (MAPK) pathway and contribute to resistance to B-Raf proto-oncogene (BRAF) and MAPK kinase (MEK) inhibitors (*51*). Accordingly, PHF8 modulation or TGFβ inhibition may limit the development of treatment resistance when combined with MAPK inhibitors.

Aside from a potential impact on response to targeted therapies, the epigenetic regulation of the TGFβ pathway by PHF8 could significantly improve response to checkpoint inhibitors. It has long been established that TGFβ secretion from tumor cells represses the production of cytolytic and proapoptotic factors by CD8^+^ cytotoxic T lymphocytes (*52*). Moreover, recent studies elegantly demonstrated that high TGFβ signaling in metastatic tumor margins contributes to reduced immune surveillance and poor therapy response in metastatic colorectal (*53*) and urothelial (*54*) cancers. The precise role of the PHF8-TGFβ signaling axis in immune infiltration of melanoma tumors and response to immunotherapy merits further examination. Our data suggest that interfering with PHF8 expression might improve response to checkpoint blockade by inhibiting TGFβ signaling, yielding better clinical outcomes in melanoma patients with tumors refractory to programmed cell death protein 1 (PD1) inhibition.

In summary, we report a mechanism of epigenetic regulation of the TGFβ pathway by PHF8 in melanoma cells that specifically governs melanoma metastasis. Our findings reveal new avenues for therapeutic intervention to improve patient outcomes.

## METHODS

### Cell culture

Cell lines were grown at 37°C in an atmosphere of 5% CO_2_. Cell lines were obtained from American Type Culture Collection, unless otherwise stated. Human embryonic kidney (HEK) 293T cells were maintained in Dulbecco’s modified Eagle’s medium (DMEM) medium (Invitrogen) containing 10% (v/v) fetal bovine serum (FBS) and 1% (v/v) penicillin/streptomycin. SKMEL-147 [a gift of A. Houghton, Memorial Sloan Kettering Cancer Center (MSKCC)], 501Mel (a gift of R. Halaban, Yale Medical School), A375, and 113/6-4L (gifts of R. Kerbel, University of Toronto) cells were cultured in DMEM medium (Invitrogen) containing 10% (v/v) FBS (Corning) and 1% (v/v) penicillin/streptomycin (Invitrogen). Colo-679 cells were cultured in RPMI 1640 medium (Invitrogen) containing 10% (v/v) FBS, 1 mM sodium pyruvate, and 1% (v/v) penicillin/streptomycin. 451Lu cells (Rockland Scientific) were cultured in TU2% medium containing 80% (v/v) MCDB153 (Sigma-Aldrich) supplemented with 1.2 g/liter NaHCO_3_ (Sigma-Aldrich), 20% (v/v) Leibovitz’s L-15 (Invitrogen), 2% (v/v) FBS, bovine insulin (5 μg/ml; Sigma-Aldrich), 1.68 mM CaCl_2_ (Sigma-Aldrich), and 1% (v/v) penicillin/streptomycin. Short Term Repeat (STR) profiling has been performed and authenticated 451Lu, 501Mel, A375, and SKMEL-147 cells. 113/6-4L is a highly metastatic variant of the WM239A human melanoma cell line (*15*). Cells were maintained in culture for no more than 20 passages and were routinely tested as negative for mycoplasma contamination.

### Data mining of human transcriptomics datasets

The log fold changes in gene expression between metastases and primaries or nevi were calculated in the following four Affymetrix transcriptomic datasets: Riker *et al.* (GSE7553; 14 primary and 40 metastatic melanomas), Talantov *et al.* (GSE3189; 18 nevi and 45 melanomas), Xu *et al.* (GSE8401; 31 primary and 52 metastatic melanomas), and Kabbarah *et al.* (GSE46517; 31 primary and 73 metastatic melanomas). Statistical comparisons were made by Student’s *t* testing. Significant differences in gene expression were considered when *P* < 0.05.

### Viral production

HEK293T cells (3 × 10^6^) were seeded per 10-cm tissue culture dish and cotransfected with lentiviral expression constructs (12 μg), viral packaging plasmid (pSPAX2, 8 μg), and viral envelope plasmid (pMD2.G, 4 μg) using Lipofectamine 2000 (Invitrogen), following the manufacturer’s recommendations. The viral supernatant was collected 48 hours after transfection, filtered through 0.45-μm filters, and stored at −80°C for long-term storage.

### Viral transduction

Target cells were seeded, incubated overnight before infection, and transduced at 30% of cell confluence. Medium was replaced with 1:4 diluted viral supernatant and Polybrene (4 μg/ml; EMD Millipore) and incubated for 6 hours, followed by replacement with growth medium. Cells were checked for fluorescent protein expression or added drug selection agents on subsequent days to ensure pure populations of transduced cells.

### Quantitative real-time PCR

RNA was extracted using the RNeasy mini kit (Qiagen) and following the manufacturer’s recommendations. Eluted RNA was quantified by Nanodrop 2000 or Qubit (Thermo Fisher Scientific) following the manufacturer’s recommendations and stored at −80C. One microgram of RNA was reverse-transcribed using the MultiScribe Reverse Transcriptase Kit (Applied Biosystems) with random hexamers (Invitrogen) following the manufacturer’s recommendations. Transcripts were quantified by qRT-PCR using Power SYBR Green qPCR MasterMix (Invitrogen). Cycle threshold values were normalized to those of the housekeeping gene GAPDH (glyceraldehyde phosphate dehydrogenase). The average for three biological replicates was plotted as relative transcript abundance. All reactions were performed in triplicate using Biorad CFX 384 or CFX 96 real-time cyclers. All primer sequences are listed in table S2.

### Plasmid preparation

All plasmid constructs were propagated in Stbl3 (Thermo Fisher Scientific) or XL-1 Blue Ultracompetent bacteria (Agilent Technologies) on LB plates or in LB media with appropriate antibiotics. Plasmids were extracted by mini- or maxi-prep (Qiagen) following the manufacturer’s recommendations. All cloned constructs were verified by Sanger sequencing before use.

#### Overexpression constructs

Retroviral constructs expressing PHF8 WT or the mutant forms PHF8 F279S and PHF8 Y14A/W29A were subcloned into lentiviral constructs with puromycin resistance.

#### sgRNA cloning

An optimal gRNA target sequence closest to the genomic target site was chosen using the http://crispr.mit.edu/ design tool. Four sgRNAs per gene that predict the best scores and lowest number of potential off-targets in exonic regions were chosen. All sgRNA sequences validated and used are listed in the table S2. The sgRNA oligonucleotides (Integrated DNA Technologies) were resuspended in annealing buffer [10 mM tris (pH 7.5 to 8.0), 50 mM NaCl, and 1 mM EDTA], mixed in equimolar concentrations, and annealed by incubation at 95°C for 5 min, followed by a slow cooling to room temperature. Annealed oligonucleotides were cloned using Bbs I (New England Biolabs) sites downstream of the human U6 promoter in a lentiviral vector containing enhanced green fluorescent protein downstream of the human PGK promoter (pLKO-sgRNA-GFP; a gift of the Brown laboratory, Mount Sinai School of Medicine, NY). Lentiviral vectors were produced as above. Melanoma cell lines stably expressing Cas9 were generated by infection with the lentiCas9-Blast (Addgene, catalog no. 52962) or Lenti-dCas9-KRAB-Blast (Addgene, catalog no. 89567) lentiviral plasmid, followed by selection with blasticidin (10 μg/ml). Cells were then infected with pLKO-sgRNA-GFP. Cells were transduced at more than 95% efficiency, and efficient knockout was assessed by Western blot 4 to 5 days after transduction.

#### shRNA constructs

plkO.1 plasmids carrying shRNA targeting human CBX2/4/8, PCGF2, CHD3, PHF8 (Sigma-Aldrich), and a nontargeting control (Dharmacon) were purchased (table S3). Lentiviral vectors and transduction were performed as detailed above.

### SMAD luciferase reporter assay

SKMEL-147 cells transduced with sgScr or sgRNAs targeting PHF8 were infected with SMAD binding element (SBE) luciferase reporter lentiviral particles (BPS Biosciences) in white opaque 96-well plates. Two days later, the luminescence assay was performed using the One-Step Luciferase Assay System (BPS Biosciences) according to the manufacturer’s instructions. In an independent set of experiments, 24 hours after SBE reporter transduction, cells were serum-deprived overnight, followed by treatment with TGFβ (10 ng/ml) or galunisertib (10 μM) for 12 hours, before measurement of SMAD activity by luciferase assay.

### In vitro invasion assay

Cell invasion was measured using 24-well Fluoroblok transwell inserts (Becton Dickinson, 8 μm pore). Briefly, inserts were coated for 2 hours at 37°C with Matrigel (Becton Dickinson/Corning) diluted in coating buffer [0.01 M tris-HCl (pH 8) and 0.7% NaCl]. For invasion experiments using cells treated with TGFβ or TGFβ inhibitor, inserts were coated with fibronectin (10 μg/ml; Becton Dickinson) to circumvent the effect of potential traces of TGFβ in Matrigel. Cells were harvested, counted in triplicate, washed, and resuspended in serum-free growth medium. Melanoma cells (20,000 to 30,000 cells per condition) were seeded per coated Fluoroblok inserts and corresponding control wells in cell input plate. Cells were allowed to settle for 10 min, followed by addition of complete growth media to the lower chamber, as chemoattractant. Twelve to 16 hours after seeding, invading cells were post-stained with Calcein AM (Thermo Fisher Scientific) diluted to 1 μg/ml in prewarmed 1× Hanks’ balanced salt solution (HBSS) for 30 min. For each independent experiment, three inserts per condition were used. Six random fields per insert were imaged using a 10× objective on an inverted fluorescence microscope. Invading cells were counted using the ImageJ software. The average of cell counts from four inserts per condition was used for plotting results.

Cell input control wells were fixed for 15 min with 1% glutaraldehyde diluted in 1× phosphate-buffered saline (PBS), washed twice with 1× PBS, and stained with a 0.5% crystal violet solution for 30 min to 2 hours at room temperature, followed by extensive washing with diH_2_O. Cells were destained with 15% acetic acid and quantified by absorbance at 595 nm. Counts of invading cell for each well were normalized to the mean absorbance of the corresponding condition from the cell input plate to control for variations in cell seeding. Only experiments with minimal variations in cell seeding between different conditions were considered.

### Western blots

Protein lysates were generated using radioimmunoprecipitation assay (RIPA) buffer (Thermo Fisher Scientific) supplemented with protease inhibitors (cOmplete EDTA-free, Roche) and phosphatase inhibitors (PhosStop, Roche) for 20 min on ice, followed by centrifugation for 15 min at 13,000 rpm at 4°C. The protein-containing supernatant was transferred to fresh microcentrifuge tubes and stored at −20° or − 80°C until further use. Protein was quantified using DC Protein Assay (Bio-Rad) following the manufacturer’s recommendations, with standard curves generated with bovine serum albumin (BSA; Sigma-Aldrich). For H4K20me1 and H4 Western blots, histone extraction was performed with the Epiquik Total Histone Extraction Kit (EpiGentek), following the manufacturer’s recommendations. Protein was quantified using Bradford Protein Assay (Bio-Rad).

Ten micrograms or 20 μg of total protein lysate was loaded per lane of 4 to 20% bis-tris polyacrylamide mini gels (Invitrogen). SDS–polyacrylamide gel electrophoresis was run at 150 V for 1.5 to 2 hours. Proteins were transferred to nitrocellulose or polyvinylidene difluoride membranes by wet transfer for 90 min at 100 V. Membranes were briefly washed once in diH_2_O, followed by blocking with 5% nonfat dry milk (Bio-Rad) or 5% BSA in tris-buffered saline supplemented with Tween 20 (0.1%) (TBS-T) for 60 min at room temperature. After blocking, membranes were washed briefly with TBS-T and incubated on a plate shaker overnight at 4°C or for 1 hour at room temperature with primary antibodies diluted in TBS-T or 5% BSA/TBS-T. Membranes were washed with TBS-T, followed by incubation with appropriate horseradish peroxidase–conjugated secondary antibodies diluted in TBS-T + 1 to 2% nonfat dry milk for 30 to 60 min at room temperature on a plate shaker. Membranes were washed extensively with TBS-T. Signal was detected using Luminata Crescendo detection system (EMD Millipore) following the manufacturer’s recommendations. All antibodies used are listed in table S1.

### Immunohistochemistry

PHF8 immunohistochemistry was performed on formalin-fixed, paraffin-embedded slides. Samples were deparaffinized, and heat-induced epitope retrieval was performed using a 1100-W microwave oven and a Nordic Ware pressure cooker filled with enough epitope retrieval solution to cover the slides. The epitope retrieval solution contains Tween, 10 mM tris, 1 mM EDTA, and 0.05% Tween 20; the pH of the solution was adjusted with HCl to pH 9.0. We use VECTASTAIN ABC, Vector Laboratories, following the manufacturer’s protocol, and a rabbit polyclonal antibody raised against mouse/human PHF8 at 1:1000 dilution. The slides were counterstained with hematoxylin and permanent-mounted with Permount. Samples were provided by the Biospecimen Core of the NYU Langone Health Interdisciplinary Melanoma Collaborative Group (IMCG). The study protocol was approved by the NYU Institutional Review Committee. All NYU patients signed informed consent. The slides were reviewed and scored by an IMCG pathologist (F.D.) according to the intensity (0, 1+ or 2+) of the staining as well as distribution (percentage of tumor with positive staining; focal: *F* < 50%; diffuse: *D* ≥ 50%). The IMCG pathologist was blinded while scoring the samples.

### Enzyme-linked immunosorbent assay

To quantitatively determine the amount of TGFB1 produced by melanoma cell lines, the Quantikine Human TGF-β1 ELISA kit (R&D Systems) was used according to the kit instructions. A representative experiment in using four technical replicates is shown out of three independent experiments.

### In vitro proliferation assay

Transduced cells were seeded at 2 × 10^3^ cells per well in 96-well plates, with the aim of fixing one plate per day for up to 5 days after seeding. The next day (day 0) and every 24 hours, cells seeded were fixed in 0.1% glutaraldehyde and stored in PBS at 4°C. At completion of the experiment, cells were stained with 0.5% crystal violet, washed, and left to dry before being dissolved with 15% acetic acid. Optical density was read at 590 nm. For normalization and control purposes, all conditions of an experiment were seeded on the same plate per day.

### In vivo metastasis

In vivo mouse experiments were performed in compliance with a referenced protocol (160719-03) approved by the NYU Institutional Animal Care and Use Committee. Four- to six-week-old nonobese diabetic (NOD)/Shi-scid/IL-2Rγ null (NOD.Cg-Prkdc^scid^Il2rg^tm1Wjl^/SzJ) female mice were purchased from The Jackson Laboratory and maintained under standard pathogen–free conditions. Experimental sample size was based on our previous experience using this xenograft model system.

451Lu cells transduced with a luciferin-expressing construct and selected with puromycin (2 μg/ml) were further transduced with Cas9 lentiviral particles and selected with blasticidin and then sgScr- or sgPHF8#1-carrying lentiviruses. For the xenograft injections, cells were resuspended in growth media at a concentration of 1 × 10^6^ cells/150 μl, aliquoted into Eppendorf tubes (150 μl), and maintained on ice until injection. Immediately before injection, cell aliquots were mixed with 150 μl of Matrigel (Becton Dickinson). Cell/Matrigel (1:1) suspensions were injected subcutaneously in the flank. No samples were excluded from the analysis. When tumors were palpable (13 days after xenograft injection), length and width measurements were made with calipers twice weekly until the animals were euthanized. When tumors were palpable, primary flank tumors were measured twice weekly by caliper [length (*l*) and width (*w*)] until resected. Tumor volumes were estimated by the formula: (*w*^2^ × *l*)/2. Forty days after subcutaneous injection, all animals were euthanized. To measure lung bioluminescence ex vivo, mice were injected with d-luciferin (Gold Biotechnologies) substrate into the intraperitoneal cavity at a dose of 150 mg/kg body weight (25 mg/ml of luciferin) 15 min before anesthesia with isoflurane/oxygen, euthanasia, and organ extraction. Lungs were placed on the imaging stage, and a critical 5-min wait between euthanasia and imaging was conserved for all animals, to ensure that luminescence can still be measured and results were comparable between mice. Images were collected by automatic exposure (0.5 s to 2 min) using In vivo imaging system (IVIS) (Xenogen Corp., Alameda, CA). Analysis was performed using Living Image software (Xenogen) by measurement of average radiance (measured in photons/s/cm^2^/steradian) with a region of interest drawn around the lung to be measured. Data were plotted using GraphPad Prism, and significance was determined by unpaired *t* test.

Macroscopic images of metastasis-bearing organs were taken with a fluorescent dissecting microscope equipped with a color camera (Leica) before fixation. Metastatic lesions were only present in the lungs. Organs (lungs, liver, kidney, ovaries, and brain) were collected, rinsed briefly in Ca_2_^+^- and Mg_2_^+^-free 1× PBS, fixed in 10% buffered formalin for 48 hours, and embedded in paraffin following standard conditions. Lung sections were sliced at three different levels, followed by hematoxylin and eosin (H&E) staining, and the metastatic foci were counted by a pathologist (F.D.) who was blinded to the denomination of samples.

### Chromatin immunoprecipitations

SKMEL-147 cells were cross-linked with 1% paraformaldehyde (in PBS 1×) for 10 min at room temperature. The reaction was quenched with 0.125 M glycine for 5 min at room temperature. After washing with PBS, cells were collected, and the pellet was lysed in 50 mM Hepes (pH 7.5), 140 mM NaCl, 1 mM EDTA, 10% glycerol, 0.5% Igepal CA-630, and 0.25% Triton X-100, freshly supplemented with 1× Protease Inhibitor Cocktail (Sigma-Aldrich). After centrifugation, isolated nuclei were washed once in 10 mM tris (pH 8.0), 200 mM NaCl, 1 mM EDTA, 0.5 mM EGTA, and 1× Protease Inhibitor Cocktail and then resuspended in ChIP buffer [10 mM tris (pH 8.0), 100 mM NaCl, 1 mM EDTA, 0.5 mM EGTA, 0.1% sodium deoxycholate, 0.5% *N*-lauryl sarcosine, and 1× Protease Inhibitor Cocktail] before sonication. After optimization for each cell line, sonication of SKMEL-147 nuclei was carried out for 30 cycles (30 s on and 30 s off), with high intensity at 4°C in a Bioruptor sonicator (Diagenode). Under these conditions, chromatin fragments have an average size of 200 base pairs (bp).

For each condition of the PHF8 ChIP-seq, 50 μg of chromatin was diluted in ChIP buffer, precleared with BSA-blocked Protein A Dynabeads (Invitrogen) for 1 hour at 4°C and then incubated with antibodies conjugated to BSA-blocked Protein A Dynabeads at 4°C overnight on an orbital shaker. The beads were washed seven times with RIPA buffer [50 mM Hepes (pH 7.6), 300 mM LiCl, 1 mM EDTA, 1% Igepal CA-630, and 0.7% sodium deoxycholate]. The beads were additionally washed twice with Tris-EDTA (TE) buffer [10 mM tris (pH 8.0) and 1 mM EDTA] supplemented with 200 mM NaCl. Then, beads were resuspended in 100 μl of elution buffer (100 mM sodium bicarbonate and 1% SDS) and incubated for 30 min at 65°C, with orbital shaking (1500 rpm). Eluates were collected, and each ChIP sample and 10% of the input resuspended in elution buffer were incubated for 30 min at 37°C with ribonuclease A (RNase A; 0.5 μg/ml) (Sigma-Aldrich). Last, Proteinase K (Roche) was added to each sample at a final concentration of 100 μg/ml, and cross-linking was reversed at 65°C overnight. DNA was extracted using a PCR purification kit (Qiagen) and further processed for sequencing or qPCR. ChIP-seq libraries were prepared with KAPA HyperPlus Kits (KAPA) according to the manufacturer’s instructions.

For H3K9me1 and H4K20me1 ChIP-qPCR, the protocol used for the ENCODE project was followed with slight modifications. Chromatin cross-linking was performed as described above. For ChIP, cells were lysed with nuclear lysis buffer [50 mM tris-HCl (pH 8.0), 10 mM EDTA, and 1% SDS] and diluted after sonication for 30 cycles (30 s on and 30 s off, with high intensity at 4°C) in dilution buffer [50 mM tris-HCl (pH 8.0), 0.167 M NaCl, 1.1% Triton X-100, and 0.11% sodium deoxycholate]. For each condition, 50 μg of sonicated chromatin was incubated overnight with BSA-blocked Protein A Dynabeads previously coupled for 6 hours with H3K9me1 or H4K20me1 antibodies. Subsequently, the beads were washed once with RIPA-150 [50 mM tris-HCl (pH 8.0), 0.15 M NaCl, 1 mM EDTA, 0.1% SDS, 1% Triton X-100, and 0.1% sodium deoxycholate], twice with RIPA-500 [50 mM tris-HCl (pH 8.0), 0.5 M NaCl, 1 mM EDTA, 0.1% SDS, 1% Triton X-100, and 0.1% sodium deoxycholate], twice with RIPA-LiCl [50 mM tris-HCl (pH 8.0), 1 mM EDTA, 1% Igepal CA-630, 0.7% sodium deoxycholate, and 0.5 M LiCl], and twice with TE buffer [10 mM tris-HCl (pH 8.0) and 1 mM EDTA]. Chromatin was eluted with elution buffer [10 mM tris-HCl (pH 8.0), 0.3 M NaCl, 5 mM EDTA, and 0.5% SDS], incubated with RNase A for 1 hour at 37°C, and then incubated with Proteinase K overnight at 65°C for decross-linking. DNA was extracted using a PCR purification kit (Qiagen), and qPCR was performed with the primers indicated in table S2.

ChIP-seq analysis was done using the in-house–developed subpipeline (*55*). Specifically, sequencing reads of PHF8 and inputs were aligned to reference genome hg19 using Bowtie2 (*56*) with a default parameter. Model-based analysis of ChIP-Seq (MACS) is used for peak calling with narrow peak calling mode and fold enrichment compared to the inputs is calculated (*57*). Significant differential peaks for each replicate were called separately with fold enrichment score > 1.5 and *q* > 0.05 as cutoff. A total of 6118 PHF8 targets were defined as genes with significant differential peaks overlapping with TSS regions (±1 kb) in all three ChIP-seq replicates. The heatmap of PHF8 binding sites was designed using seqMINER (https://ncbi.nlm.nih.gov/pubmed/21177645).

### RNA-seq and analysis

RNA was extracted from three biological replicates using a QIAGEN RNeasy minikit. RNA quality was defined on an Agilent 2100 Bioanalyzer, then processed with the Ribo-Zero rRNA Removal Kit (Illumina) to remove ribosomal RNA (rRNA), and further processed into sequencing libraries using the Illumina ScriptSeq Complete Gold kit following the manufacturer’s protocol. All libraries were sequenced on Illumina HiSeq2500 (~150 M, 50 bp paired-end) with individual samples spread across multiple sequencing lanes. Indexed sample data were demultiplexed, and individual FASTQ files were generated, followed by quality control assessment with FASTQC. RNA-seq analysis was done using the in-house–developed subpipeline (*55*). Specifically, sequencing reads of sgRNA knockdown samples and their control samples aligned to reference genome hg19 using STAR-aligner version 2.4.2 (*58*) with parameters suggested by TCGA expression mRNA-seq pipeline (https://docs.gdc.cancer.gov/Data/Bioinformatics_Pipelines/Expression_mRNA_Pipeline/), and the raw read counts were generated. Then, DESeq2 was used to perform differential expression analysis between sgRNA samples and control samples (*59*). The volcano plot was generated by R. Genes with *P* < 0.05 and log_2_ fold change lower than −0.25 and higher than 0.25 were considered significantly differentially expressed. Fold change and *P* value of differentially expressed gene sets have been imported into QIAGEN’s Ingenuity Pathway Analysis (IPA QIAGEN Redwood City, http://ingenuity.com) for pathway analyses, setting a log_2_ fold change lower than −0.3 and higher than 0.3 as cutoff. Area proportional Venn diagrams were generated with BioVenn (https://ncbi.nlm.nih.gov/pubmed/18925949).

### Statistical analyses

Statistical analyses were performed with GraphPad Prism (GraphPad Software Inc.). Data are presented as the means ± SD. Significance was determined using unpaired/paired Student’s *t* test, Mann-Whitney test, or log-rank test (Kaplan-Meier curves), where appropriate. The statistical analyses were performed, and *P* values were indicated in each figure legend. Correlations were analyzed by Spearman correlation in GraphPad Prism. *P* values are represented as *P < 0.05, **P < 0.01, ***P < 0.001, ****P < 0.0001, and *****P < 0.00001.

## Notes

View/request a protocol for this paper from *Bio-protocol*.
