# Supplementary material for: The histone demethylase PHF8 regulates TGFβ signaling and promotes melanoma metastasis
Source: Sci Adv. 2022 Feb 18;8(7):eabi7127. doi: 10.1126/sciadv.abi7127 (PMC8856617; doi:10.1126/sciadv.abi7127)
Supplement: Supplementary file 1 — Figs. S1 to S6 [file sciadv.abi7127_sm.pdf]

Science Advances
AAAS

Supplementary Materials for

The histone demethylase PHF8 regulates TGFβ signaling and promotes melanoma metastasis

Rana S. Moubarak*, Ana de Pablos-Aragoneses, Vanessa Ortiz-Barahona, Yixiao Gong, Michael Gowen, Igor Dolgalev, Sorin A. A. Shadaloey, Diana Argibay, Alcida Karz, Richard Von Itter, Eleazar Carmelo Vega-Sáenz de Miera, Elena Sokolova, Farbod Darvishian, Aristotelis Tsirigos, Iman Osman, Eva Hernando*

*Corresponding author. Email: rana.moubarak@nyulangone.org (R.S.M.); eva.hernando-monge@nyulangone.org (E.H.)

Published 18 February 2022, *Sci. Adv.* **8**, eabi7127 (2022)
DOI: 10.1126/sciadv.abi7127

The PDF file includes:

Figs. S1 to S6
Legends for tables S1 to S3

Other Supplementary Material for this manuscript includes the following:

Tables S1 to S3

Supplementary Figures

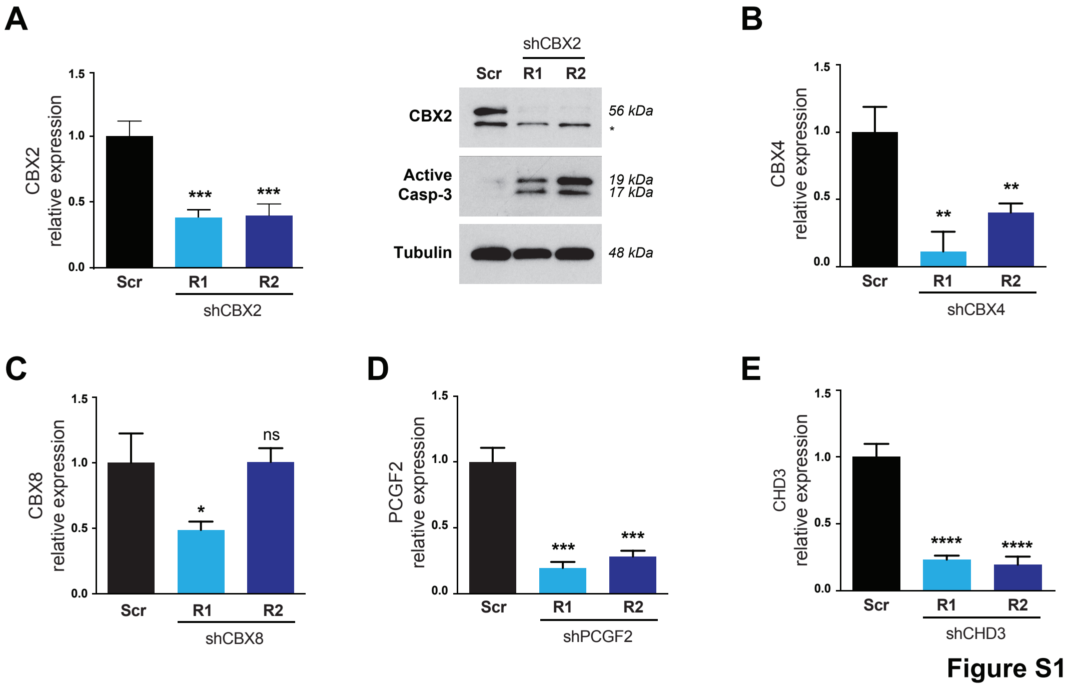

Figure S1: **Validation of knockout efficiency of genes selected for the proliferation and invasion screens.** Knockdown efficiency of two different shRNA targeting A) CBX2 (R1 vs Scr, p=0.0005; R2 vs Scr, p=0.001), B) CBX4 (R1 vs Scr, p=0.008; R2 vs Scr, p=0.009), C) CBX8 (R1 vs Scr, p=0.03; R2 vs Scr, p=0.93), D) PCGF2 (R1 vs Scr, p=0.0004; R2 vs Scr, p=0.0005) and E) CHD3 (R1 vs Scr, p=0.00002; R2 vs Scr, p=0.0001), were measured by qRT-PCR. In A) CBX2 knockdown and subsequent activation of caspase-3 were assessed by western blot. Tubulin blotting was used as loading control.

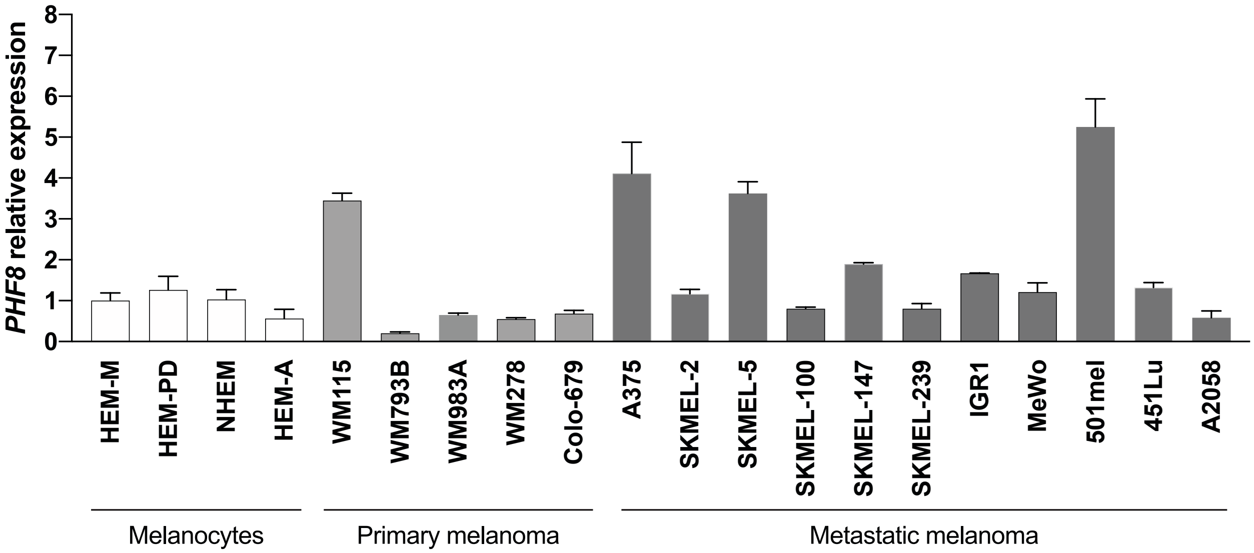

Figure S2

Figure S2: ***PHF8* mRNA expression in a panel of melanocytes, primary and metastatic melanoma cell lines** was measured by qRT-PCR.

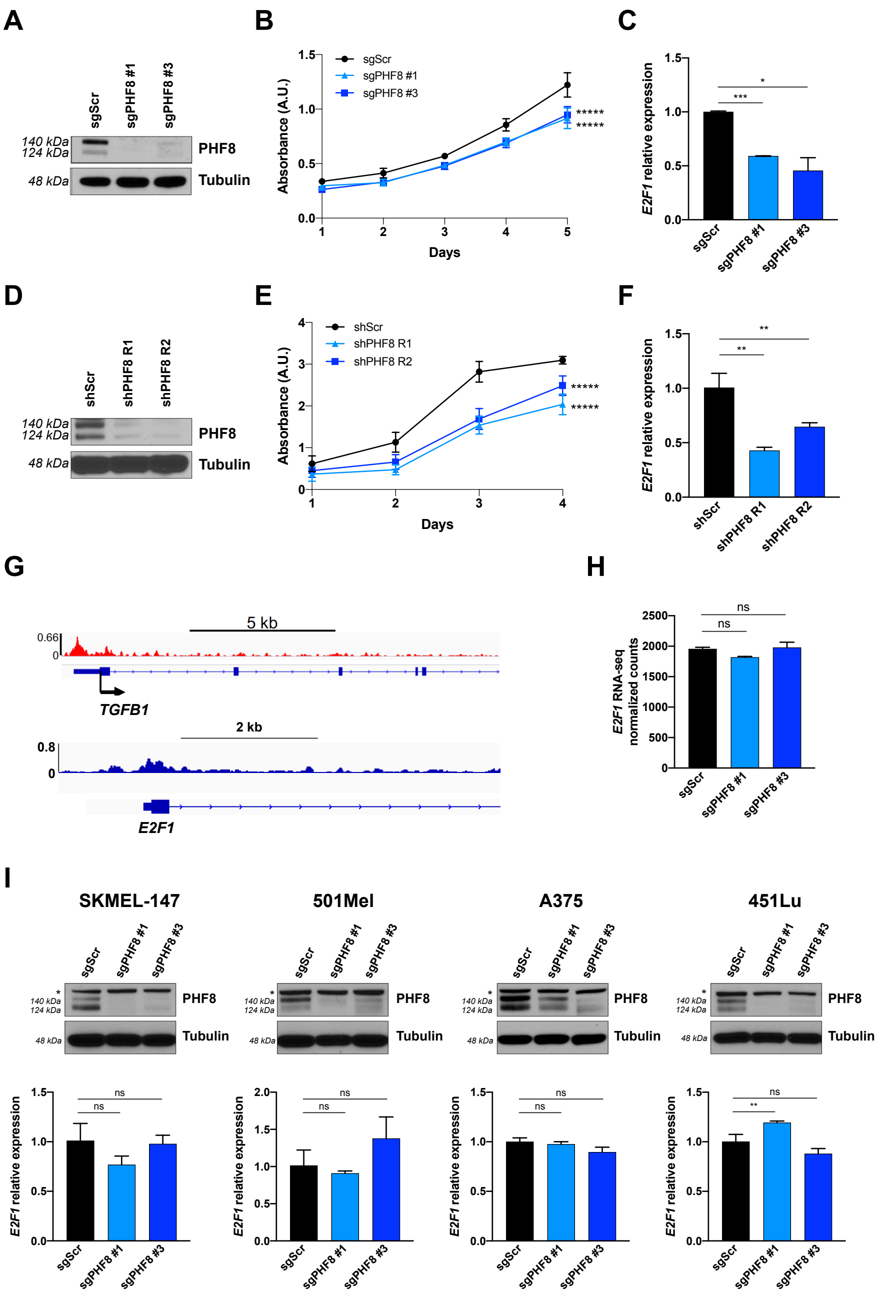

Figure S3

Figure S3: ***E2F1* is a PHF8 target in HeLa cells but not melanoma cell lines**. A) Western blot with anti-PHF8 antibody reveals efficient PHF8 depletion using the CRISPR/Cas9 system and 2 different sgRNAs in HeLa cells. B) PHF8 depletion in HeLa cells results in significantly reduced proliferation (sgPHF8 #1 vs sgScr, p<0.000001; sgPHF8#3 vs sgScr, p<0.000001), and C) downregulated *E2F1* transcriptional levels (sgPHF8 #1 vs sgScr, p=0.0002; sgPHF8#3 vs sgScr,

p=0.02). Representative data of two independent experiments are shown. D) Western blot controls for efficient PHF8 knockdown using 2 different shRNAs. E) PHF8 knockdown in HeLa cells results in significantly reduced proliferation rates (shPHF8 R1 vs sgScr, p<0.000001; shPHF8 R2 vs sgScr, p<0.000001), and F) *E2F1* transcriptional levels (shPHF8 R1 vs sgScr, p=0.0017; shPHF8 R2 vs sgScr, p=0.01). Tubulin reblotting serves as loading control. G) In SKMEL-147 melanoma cells, PHF8 ChIP-seq analyses reveal that, unlike *TGFB1*, *E2F1* is not a PHF8 target, as it did not reach MACS2 significance. Red and blue PHF8 ChIP-seq peaks illustrate statistically significant and non-significant peaks, respectively. H) RNA-seq analyses of SKMEL-147 upon PHF8 depletion show that PHF8 is not a transcriptional regulator of *E2F1* in melanoma (sgPHF8 #1 vs sgScr, p=0.1; sgPHF8 #3 vs sgScr, p=0.7). I) PHF8 depletion in 4 melanoma cell lines does not significantly downregulate *E2F1* levels: SKMEL-147 (sgPHF8 #1 vs sgScr, p=0.09; sgPHF8 #3 vs sgScr, p=0.78), 501mel (sgPHF8 #1 vs sgScr, p=0.43; sgPHF8 #3 vs sgScr, p=0.15), A375 (sgPHF8 #1 vs sgScr, p=0.40; sgPHF8 #3 vs sgScr, p=0.07) and 451Lu (sgPHF8 #1 vs sgScr, p=0.01; sgPHF8 #3 vs sgScr, p=0.07).

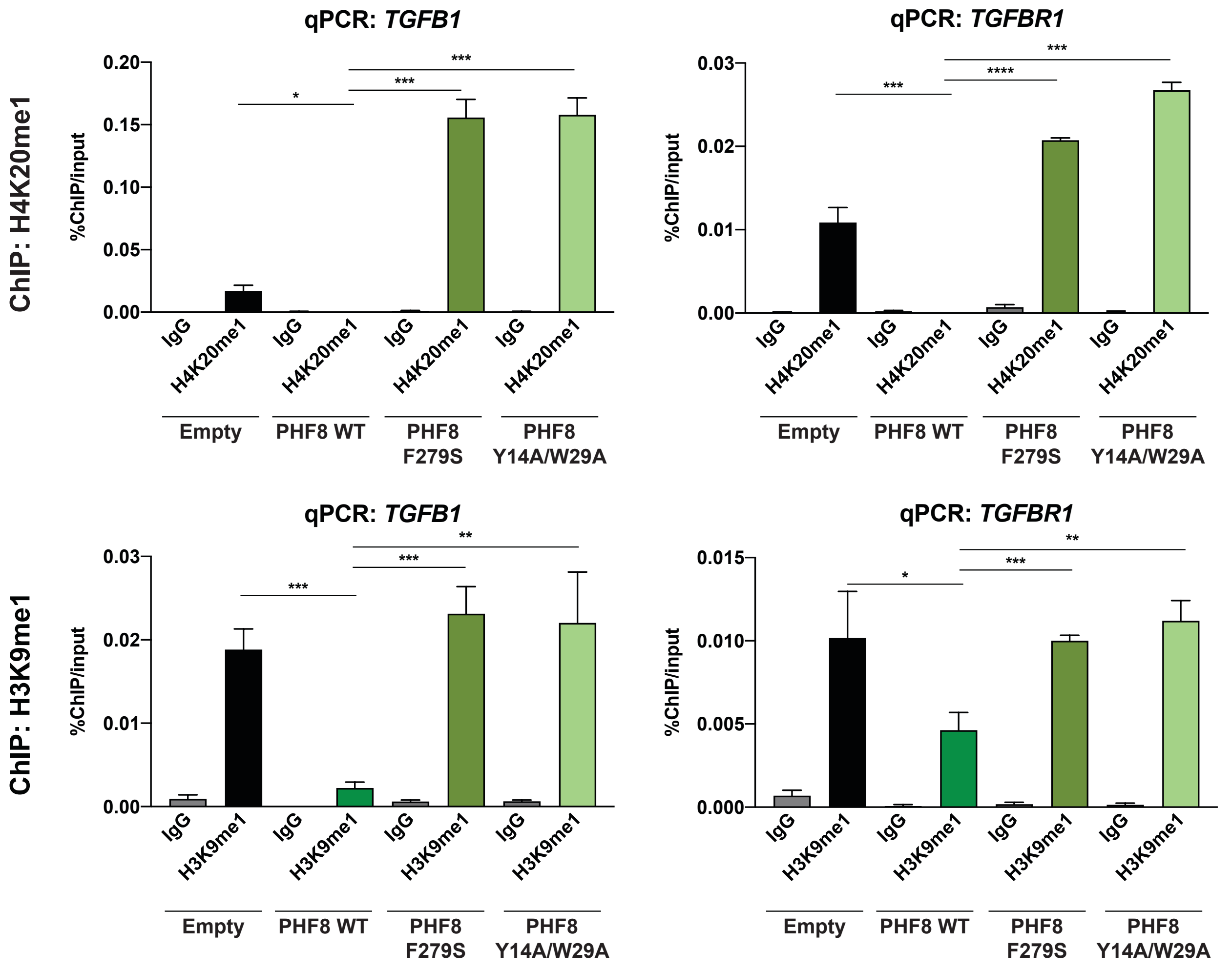

Figure S4

Figure S4: Overexpression of PHF8 WT, but not of F279S and Y14A/W29A mutants, suppresses H4K20me1 and H3K9me1 deposition at the TSS regions of *TGFB1* and *TGFBR1*. H4K20me1 and H3K9me1 ChIP experiments followed by qPCR of *TGFB1* and *TGFBR1* regions bound by PHF8 were performed in 113/6-4L cells transduced with Empty, PHF8 WT, PHF8 F279S or PHF8 Y14A/W29A lentiviruses: H4K20me1 ChIP and *TGFB1* qPCR (PHF8 WT vs Empty, $p=0.018$; PHF8 F279S vs PHF8 WT, $p=0.0007$; PHF8 Y14A/W29A vs PHF8 WT, $p=0.0006$), H4K20me1 ChIP and *TGFBR1* qPCR (PHF8 WT vs Empty, $p=0.0005$; PHF8 F279S vs PHF8 WT, $p<0.000001$; PHF8 Y14A/W29A vs PHF8 WT, $p=0.0002$), H3K9me1 ChIP *TGFB1* qPCR (PHF8 WT vs Empty, $p=0.0004$; PHF8 F279S vs PHF8 WT, $p=0.0004$; PHF8 Y14A/W29A vs PHF8 WT,

p=0.005), H3K9me1 ChIP *TGFBR1* qPCR (PHF8 WT vs Empty, p=0.03; PHF8 F279S vs PHF8 WT, p=0.001; PHF8 Y14A/W29A vs PHF8 WT, p=0.002).

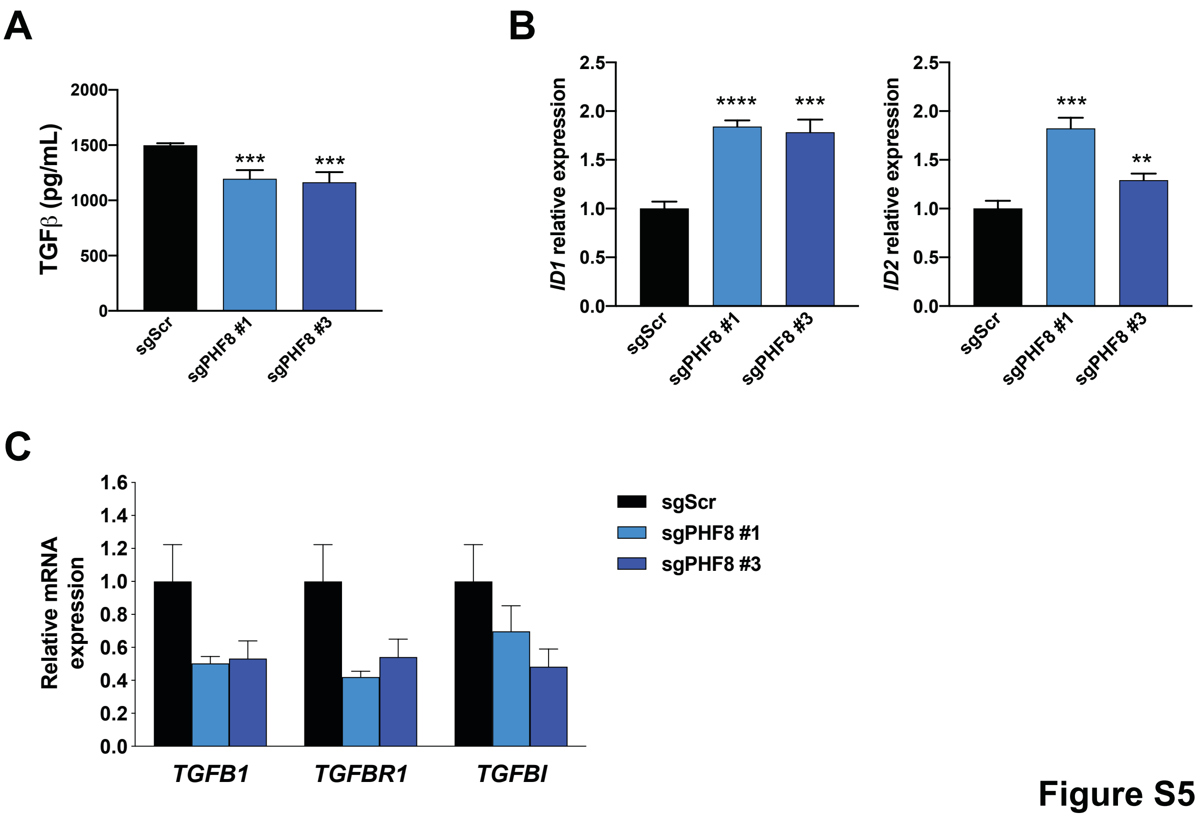

Figure S5: PHF8 controls TGFβ secretion and the expression of *TGFβ* genes and targets. A) TGFβ1 production was assessed by ELISA in conditioned media of SKMEL-147 cells transduced with sgScr, sgPHF8#1 or sgPHF8#3 and cultured for 5 days. PHF8 depletion significantly reduces TGFβ1 secretion by melanoma cells (sgPHF8 #1 vs sgScr, p=0.0003; sgPHF8 #3 vs sgScr, p=0.0004). B) PHF8 knock-out results in upregulation of TGFβ target genes *ID1* (sgPHF8 #1 vs sgScr, p=0.0001; sgPHF8 #3 vs sgScr, p=0.0007) and *ID2* (sgPHF8 #1 vs sgScr, p=0.0004; sgPHF8 #3 vs sgScr, p=0.008), as measured by qRT-PCR in SKMEL-147 cells transduced with sgScr, sgPHF8#1 or sgPHF8#3. C) Downregulation of *TGFB1*, *TGFBR1* and *TGFBI* mRNAs upon PHF8 knockout in SKMEL-147 was measured by qRT-PCR.

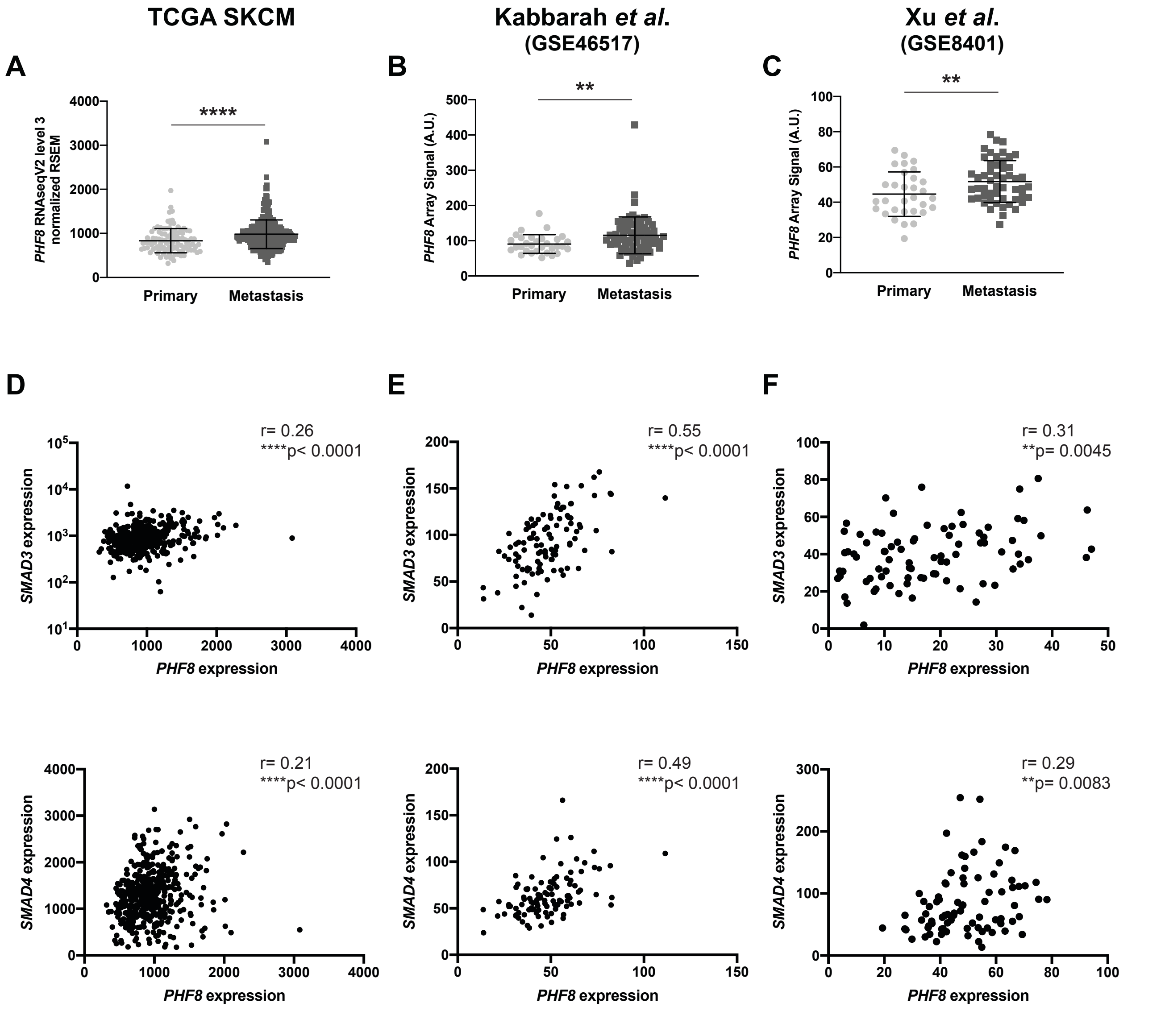

Figure S6

Figure S6: *PHF8* levels positively correlate with the expression of its transcriptional targets in multiple transcriptomic datasets. Dot plots of *PHF8* expression showing a significant upregulation of *PHF8* expression in metastatic versus primary melanoma tumors in **A**) TCGA SKCM dataset (p<0.0001), **B**) Kabbarah et al. (p=0.0029) and **C**) Xu et al. transcriptomic datasets (p=0.0140)

(Mann-Whitney test). *PHF8* expression positively correlates with *SMAD3* and *SMAD4* expression in **D**) TCGA, **E**) Kabbarah et al. and **F**) Xu et al. transcriptomic datasets. Spearman regression coefficient (r) and p-values are indicated for each graph.

Supplementary Table 1: Table related to Fig. 1B representing the 151 chromatin-related genes found upregulated in metastatic versus primary melanoma in at least 2 out of the 4 transcriptomic datasets mined. The six genes chosen for this study are labeled in red. A coding system was used to generate the illustrative table in Fig. 1B, using green for genes significantly upregulated in metastatic versus primary samples ($p<0.05$), yellow for genes significantly downregulated in metastatic versus primary samples ($p<0.05$) and white for genes that show no significant changes in expression.

Supplementary Table 2: Table related to Fig. 1B representing the LogFC of transcriptional changes in metastatic versus primary melanoma (Riker *et al*. (8), Xu *et al*. (10) and Kabbarah *et al*. (7)) and the LogFC in melanoma versus nevi (Talantov *et al*. (9)) for the six genes selected for functional screens.

Supplementary Table 3: Tables of reagents used in the manuscript. Lists of antibodies, commercial kits, oligonucleotides sequences, and plasmids.
