# Supplementary material for: The histone demethylase PHF8 regulates TGFβ signaling and promotes melanoma metastasis
Source: Sci Adv. 2022 Feb 18;8(7):eabi7127. doi: 10.1126/sciadv.abi7127 (PMC8856617; doi:10.1126/sciadv.abi7127)
Supplement: Supplementary file 2 — Tables S1 to S3 [file sciadv.abi7127_tables_s1_to_s3.zip › sciadv.abi7127_table_s3.xlsx]

Supplementary information: Reagents

Table 1. Antibodies, Reagents and Kits

Antibody	Manufacturer
CBX2	Abcam
Cleaved Caspase-3 (Asp175) Antibody	Cell Signaling Technology
FLAG-M2	Sigma
H3K9me1	Abcam
H4K20me1	Abcam
HA	Santa Cruz
Rabbit IgG	Bethyl
PHF8	Abcam
P-SMAD2 (S465/467)	Cell Signaling Technology
SMAD2/3	Cell Signaling Technology
Tubulin	Sigma
Actin-peroxidase conjugated antibody	Sigma
Anti-Rabbit IgG (whole molecule)–Peroxidase antibody produced in goat	Sigma
Anti-Mouse IgG (whole molecule)–Peroxidase antibody produced in rabbit	Sigma
Epiquik Total Histone Extraction Kit	Epigentek
SBE Luciferase Reporter Lentivirus (TGFbeta/SMAD Pathway	BPS Biosciences
ONE-Step Luciferase Assay System	BPS Biosciences
Quick Start Bradford Protein Assay Kit	Bio-Rad
DC Protein Assay kit	Bio-Rad

Table 2. Oligonucleotides (IDT DNA)

qRT-PCR primers	Sequence
CBX2 Forward	5'-GGCTGGTCCTCCAAACATAAC-3'

CBX2 Reverse	5’-TCTGCACCTCCTTCTCATGT-3’
CBX4 Forward	5’-GCAGAGTGGAGTATCTGGTGAA-3’
CBX4 Reverse	5’-TTCCCTGTTCTGGAAGGCGAT-3’
CBX8 Forward	5’-CAACATGGAGCTTTCAGCGG-3’
CBX8 Reverse	5’-ATTCCATGCGTCCTTTCCGT-3’
PCGF2 Forward	5’-CGCAACAAGATGGATGTGCC-3’
PCGF2 Reverse	5’-TAGGCGATGTCCATGAGGGT-3’
CHD3 Forward	5’-GCGAAAGCTGAAGGAGCAAG-3’
CHD3 Reverse	5’-ACCATCGATGCGCTCATACTT-3’
PHF8 Forward	5’-GAGAACAGGAGACACCCTGC-3’
PHF8 Reverse	5’-AATGAGCTGTACGGTTCGCA-3’
TGFB1 Forward	5’-ACAGCAACAATTCCTGGCGA-3’
TGFB1 Reverse	5’-GAACCCGTTGATGTCCACTTG-3’
TGFBR1 Forward	5’-CAACGTCAGGTTCTGGCTCA-3’
TGFBR1 Reverse	5’-ACAGCAACTTCTTCTCCCCG-3’
TGFBR2 Forward	5’-GCACGTTCAGAAGTCGGATG-3’
TGFBR2 Reverse	5’-CTGCACCGTTGTTGTCAGTG-3’
TGFBI Forward	5’-TGCTCCCACAAATGAAGCCT-3’
TGFBI Reverse	5’-GCCTCCGCTAACCAGGATTT-3’
ID1 Forward	5’-AATCCGAAGTTGGAACCCCC-3’
ID1 Reverse	5’-AACGCATGCCGCCTCG-3’
ID2 Forward	5’-CTGCAGCACGTCATCGACTA-3’
ID2 Reverse	5’- TTCAGAAGCCTGCAAGGACA-3’
GAPDH Forward	5’-CGCTCTCTGCTCCTCCTGTT-3’
GAPDH Reverse	5’-CCATGGTGTCTGAGCGATGT-3’
**ChIP-qPCR primers**	**Sequence**
gTGFB1, Forward	5’-GAGGGCTGGTCCGGAATG-3’

gTGFB1, Reverse	5’-GAGACTTTTCCGTTGCCGC-3’
gTGFBR1, Forward	5’-GAAAAGAGCGTCGAACGGC-3’
gTGFBR1, Reverse	5’-GGGTAGAGCGATGGGTGTGT-3’
gTGFBR2, Forward	5’-CACGTTCAGAAGTCGGGTGA-3’
gTGFBR2, Reverse	5’-TCACAATCCCTGCAGCTACG-3’
**sgRNA oligonucleotides**	**Sequence**
sgRNA Non-Targeting Control sequence, Forward (sgScr)	5’-caccgCGCTTCCGCGGCCCGTTCAA-3’
sgRNA Non-Targeting Control sequence, Reverse (sgScr)	5’-aaacTTGAACGGGCCGCGGAAGCGc-3’
sgRNA sequence targeting PHF8, Forward (sgPHF8 #1)	5’- caccGTCACACTCGATCATGAAGC-3’
sgRNA sequence targeting PHF8, Reverse (sgPHF8 #1)	5’-aaacGCTTCATGATCGAGTGTGAC-3’
sgRNA sequence targeting PHF8, Forward (sgPHF8 #3)	5’-caccgTCCTCCTGAGCCTAAACAAG-3’
sgRNA sequence targeting PHF8, Reverse (sgPHF8 #3)	5’-aaacCTTGTTTAGGCTCAGGAGGAc-3’
sgRNA sequence targeting TGFBR2, Forward (sgTGFBR2 #A)	5’-caccGGCAGCTACGAGAGAGCTAG-3’
sgRNA sequence targeting TGFBR2, Reverse (sgTGFBR2 #A)	5’-aaacCTAGCTCTCTCGTAGCTGCC-3’
sgRNA sequence targeting TGFBR2, Forward (sgTGFBR2 #E)	5’-caccGAGCTAGGGGCTGGACGTCG-3’
sgRNA sequence targeting TGFBR2, Reverse (sgTGFBR2 #E)	5’-aaacCGACGTCCAGCCCCTAGCTC-3’

Table 3. Plasmids

Plasmid	Source	Identifier
pLenti-Cas9-Blast	Addgene	52962 (Dr Feng Zhang's lab)
Lenti-dCas9-KRAB-Blast	Addgene	89567 (Dr Gary Hon's lab)
pLKO-sgRNA-GFP	MSSM, NY	Gift from Dr. Brian Brown
pLenti-CMV-GFP-Puro	Addgene	17448 (Dr Eric Campeau's lab)
psPAX2	Addgene	12260 (Dr Didier Trono's lab)
pMD2.G	Addgene	12259 (Dr Didier Trono's lab)
pLenti CMV Puro LUC (w168-1)	Addgene	17477 (Dr Eric Campeau's lab)
plkO.1-human-shSCR	Dharmacon	RHS6848
plkO.1-human-shCBX2 R1	Sigma	TRCN0000378282
plkO.1-human-shCBX2 R2	Sigma	TRCN0000368640
plkO.1-human-shCBX4 R1	Sigma	TRCN0000234579
plkO.1-human-shCBX4 R2	Sigma	TRCN0000234580
plkO.1-human-shCBX8 R1	Sigma	TRCN0000363346
plkO.1-human-shCBX8 R2	Sigma	TRCN0000363282
plkO.1-human-shPCGF2 R1	Sigma	TRCN0000280911
plkO.1-human-shPCGF2 R2	Sigma	TRCN0000280912
plkO.1-human-shCHD3 R1	Sigma	TRCN0000107974
plkO.1-human-shCHD3 R2	Sigma	TRCN0000107971
plkO.1-human-shPHF8 R1	Sigma	TRCN0000358911
plkO.1-human-shPHF8 R2	Sigma	TRCN0000358845
pOZ-N-FLAG-HA-Empty	Children's Hospital, Harvard	Gift from Dr. Yang Shi
pOZ-N-FLAG-HA-PHF8 WT	Children's Hospital, Harvard	Gift from Dr. Yang Shi
pOZ-N-FLAG-HA-PHF8 F279S	Children's Hospital, Harvard	Gift from Dr. Yang Shi

pOZ-N-FLAG-HA-PHF8 Y14A/W29A	Children’s Hospital, Harvard	Gift from Dr. Yang Shi
pLenti-Puro-Empty	Cloned for this paper	N/A
pLenti-Puro-PHF8 wt	Cloned for this paper	N/A
pLenti-Puro-PHF8 F279S	Cloned for this paper	N/A
pLenti-Puro-PHF8 Y14A/W29A	Cloned for this paper	N/A
